# Selection and Validation of Optimal RT-qPCR Reference Genes for the Normalization of Gene Expression under Different Experimental Conditions in *Lindera megaphylla*

**DOI:** 10.3390/plants12112185

**Published:** 2023-05-31

**Authors:** Hongli Liu, Jing Liu, Peng Chen, Xin Zhang, Ke Wang, Jiuxing Lu, Yonghua Li

**Affiliations:** 1 International Union Laboratory of Landscape Architecture of Henan, College of Landscape Architecture and Arts, Henan Agricultural University, Zhengzhou 450003, China; 2 Zhengzhou Botanical Garden, Zhengzhou 450042, China

**Keywords:** *Lindera megaphylla*, normalization genes, RT-qPCR, tissue specificity, temperature, transcript analysis

## Abstract

*Lindera megaphylla*, a broad-leaved evergreen that is used as a landscape ornamental plant and medicinal plant, is an ecologically important and dominant tree species. However, little is known about the molecular mechanisms of its growth, development, and metabolism. The selection of suitable reference genes is critical for molecular biological analyses. To date, no research on reference genes as a foundation for gene expression analysis has been undertaken in *L. megaphylla*. In this study, 14 candidate genes were selected from the transcriptome database of *L. megaphylla* for RT-qPCR assay under different conditions. Results showed that *helicase-15* and *UBC28* were most stable in different tissues of seedlings and adult trees. For different leaf developmental stages, the best combination of reference genes was *ACT7* and *UBC36*. *UBC36* and *TCTP* were the best under cold treatment, while *PAB2* and *CYP20-2* were the best under heat treatment. Finally, a RT-qPCR assay of *LmNAC83* and *LmERF60* genes were used to further verify the reliability of selected reference genes above. This work is the first to select and evaluate the stability of reference genes for the normalization of gene expression analysis in *L. megaphylla* and will provide an important foundation for future genetic studies of this species.

## 1. Introduction

*Lindera megaphylla* is a predominant, broad-leaved, and aromatic evergreen tree species belonging to the Lauraceae family and is widely distributed in the subtropical and warm-temperate zones of China. *L. megaphylla* has not only ecological and ornamental value, but also medicinal and therapeutic value as a source of an essential oils, spices, and drugs [1,2,3]. For example, d-dicentrine, an aporphine alkaloid, is isolated from the root of *L. megaphylla* and has potential antitumor activity [4,5]. These trees are rich in terpenoids, alkaloids, and flavonoids, many of which could be used to make pesticides or industrial feedstocks, while its volatile compounds, mainly terpenoids, have strong bactericidal ability. These trees can also help to improve air quality [6], a property that could be improved through molecular breeding. To synthesize antibacterial compounds, it is necessary to first explore the related regulatory genes and to analyze their functions. To date, studies of *L. megaphylla* have largely focused on the cultivation of seedlings, various kinds of biotic and abiotic stress responses and the analysis of volatile substances and their potential applications [7,8,9,10,11,12], while few studies have focused on the molecular biology of *L. megaphylla* due to a lack of genomic information. Gene expression analysis circumvents that lack of a sequenced genome to explore the molecular mechanisms underlying transcriptional regulation of phenotype. Transcriptome datasets derived from different tissues and differently aged leaves of *L. megaphylla* have been obtained (unpublished data), which will greatly promote functional genetic studies in this species.

Real-time reverse transcriptase quantitative polymerase chain reaction (RT-qPCR) is an important tool for analyzing gene expression because of its high throughput, sensitivity and precision [13,14]. However, RT-qPCR data are affected by many factors, such as extraction protocols, the purity and integrity of the extracted RNA, the efficiency of reverse transcription and PCR amplification, and primer specificity [15,16]. Therefore, a stably expressed reference gene is essential to avoid unnecessary errors generated through confounding factors and to increase the accuracy of the RT-qPCR data analysis. In the pre-genomic era, traditional reference genes were chosen based on their known or suspected housekeeping roles in basic cellular processes, cell structure, or primary metabolism, including genes encoding actin (*ACT*), 18S rRNA (*18S*) and glyceraldehyde-3 phosphate dehydrogenase (*GAPDH*) [17,18,19,20]. Unfortunately, these housekeeping genes are not stably expressed in all tissues, under different experimental conditions, or between different species [21,22]. In addition, a growing number of studies have revealed that some novel genes can also be used as internal references and are better than some traditional housekeeping genes [23,24,25,26]. Thus, it is necessary to systematically select the most appropriate reference genes to ensure the accuracy of RT-qPCR analysis for specific conditions and in specific materials. For this reason, optimal reference genes for transcript normalization must be determined through statistical algorithms such as delta cycle threshold (ΔCt) [27], geNorm [28], NormFinder [29], BestKeeper [30], and RefFinder [31] for each type of sample. These algorithms are widely used to assess the transcript stability of candidate reference genes in various species [32,33,34,35]. The use of reference genes in expression analysis has greatly facilitated our understanding of the important information related to gene functions and complex biological processes in plants, such as the signaling and metabolic pathways that underlie developmental and cellular processes [36,37,38,39].

Gene expression databases of model plant species such as *Arabidopsis thaliana* and tomato (*Lycopersicon esculentum*) are important resources for identifying and searching for genes of interest and their expression patterns (http://www.ebi.ac.uk/arrayexpress/; accessed on 5 June 2022; http://www.ncbi.nlm.nih.gov/geo/, accessed on 5 June 2022) [17]. Czechowski et al. selected, verified, and recommended 18 new reference genes that were superior to traditional reference genes in terms of expression stability across an extensive sample series or under a range of environmental conditions through the publicly available AtGenExpress database (http://web.uni-frankfurt.de/fb15/botanik/mcb/AFGN/atgenex.htm, accessed on 5 June 2022) and the author’s own ATH1 database [17]. Orthologues of known genes in Arabidopsis can serve the same purposes as in other species. In addition, Lin et al. validated suitable reference genes for reliable normalization of data from *Litsea cubeba* [40]. Moreover, both *L. megaphylla* and *L. cubeba* belong to the Lauraceae family, and their evolutionary relationship is relatively close.

Based on these previous results, we first selected 40 genes that have been used as internal reference from published model plants such as Arabidopsis thaliana and other species in the Lauraceae family as candidate reference genes for *L. megaphylla*. Second, we screened homologous sequences in the transcriptome database of *L. megaphylla* and obtained 20 candidate genes based on expression multiples less than 1.5 and FPKM value > 50 in different tissues.

In this study, 14 candidate genes were identified with E-values between 91.035% and 107.169% and R^2^ values from 0.991 to 0.999, indicating that the primer pairs may be more accurate for standardized evaluation by RT-qPCR. The candidate genes included translationally controlled tumor protein (*TCTP*), *ACT7*, *GAPDH*, ubiquitin-conjugating enzyme E2 36/7 (*UB C36*, *UBC7*), elongation factor 2-like (*EF2*), peptidyl-prolyl cis-trans isomerase CYP20-2, chloroplastic (*CYP20-2*), polyubiquitin (*UBQ*), alpha-tubulin (*TUA*), ubiquitin-conjugating enzyme E2 28-like (*UBC28*), NADH dehydrogenase (*ubiquinone*), pentatricopeptide repeat-containing protein (PPR), eukaryotic initiation factor 4A-3-like (*EIF4A-3*), DEAD-box ATP-dependent RNA helicase 15 (*helicase-15*), and polyadenylate-binding protein 2-like (*PAB2*). These 14 candidate genes were then assessed for stability of expression under specific conditions, including different tissues of one-year-old seedlings (roots, stems, and leaves), tissues of 10-year-old trees (leaf buds, young stems, young seeds, young leaves, and mature leaves), 16 different leaf developmental stages and under different temperature stresses (cold and heat). Finally, the *NAC* and *ERF* genes, which are from an important family of transcription factors in plants, were used to verify the reliability of the selected reference genes in different samples. Our research identified the best reference genes for RT-qPCR analysis of *L. megaphylla* tissues under different conditions, laying a basis for further studies of the molecular mechanisms regulating gene expression in this important tree species.

## 2. Materials and Methods

### 2.1. Plant Materials and Treatments

*L. megaphylla* trees were grown in a field at the Zhengzhou Botanical Garden located in Zhengzhou city, Henan Province, China. Young seedlings were cultivated in plant growth chambers with LED lighting for temperature treatments. In total, 6 experimental sets were cultivated for RNA extraction. The first experimental set consisted of roots, stems, and leaves from 1-year-old seedlings that displayed robust and consistent growth. The second experimental set consisted of leaf buds, young stems, young seeds, young leaves, and mature leaves from adult trees of *L. megaphylla* that had been growing in the natural environment for approximately 10 years. The third experimental set included leaves at different developmental stages. Growing leaves were collected roughly every 3–7 or 3–15 days from the beginning of leaf bud growth in late March until the leaves were fully mature by the end of July. The specific sampling dates were 24 March, 29 March, 1 April, 4 April, 7 April, 15 April, 22 April, 29 April, 14 May, 23 May, 4 June, 16 June, 1 July, 15 July, and 31 July. The fourth set was exposed to cold stress [41]. Furthermore, 1-year-old seedlings were grown at 65% relative humidity and under 16 h/8 h light/dark conditions in an LED plant growth incubator (Shengyuan Instrument Co., Ltd., Zhengzhou, China). The seedlings were first treated at 25 °C for 7 days as control, and leaves were collected. Then, the seedlings were transferred to 4 °C for 7 days for long-term chilling acclimation (CA). Next, the seedlings were shifted to 0 °C for an additional 7 days for long-term freezing acclimation (FA). Then, the seedlings were again moved to control conditions (25 °C) for 7 days for long-term de-acclimation (DA). The fifth and sixth experimental groups consisted of leaves collected from 1-year-old seedlings that were treated with cold and heat, respectively. For cold treatments, the seedlings were cultivated in an LED plant growth incubator at 25 °C, 4 °C, 0 °C, −4 °C, or −6 °C for 24 h. For heat treatments, the seedlings were cultivated in an incubator at 25 °C, 30 °C, 35 °C, 40 °C, or 45 °C for 24 h. Data regarding all six sample sets described above are summarized in Table 1. All collected samples were immediately frozen in liquid nitrogen and stored at −80 °C. Three independent biological replicates were collected for each sample.

### 2.2. Total RNA Extraction and cDNA Synthesis

Total RNA was extracted from 0.05 g samples using a Quick RNA isolation kit (HUAYUEYANG Biotechnology, Beijing, China) according to the manufacturer’s protocol [42]. The RNA integrity, purity and concentration were assessed using 2% (*w*/*v*) agarose gel electrophoresis and a NanoDrop ND-1000 spectrophotometer (Thermo Scientific, Waltham, MA, USA). Total RNA (1 μg) with A_260_/A_280_ and A_260_/A_230_ ratios greater than 1.8 was used for first-strand cDNA synthesis using the Evo M-MLV RT Kit with gDNA Clean for qPCR according to the manufacturer’s instructions (Accurate Biotechnology, Changsha, China). Specifically, a 10 μL reaction system (1 µg total RNA, 2 µL 5 × gDNA Clean Reaction Mix, to a total volume of 10 µL with RNase-free water) was subjected to 42 °C for 2 min. Then, a 20 μL reaction system (10 μL of first reaction solution, 4 μL 5 × *Evo M-MLV* RT Reaction Mix and 6 μL RNase-free water) was subjected to 37 °C for 15 min and 85 °C for 5 s. Five-fold diluted cDNA was used for subsequent RT-qPCR experiments. All cDNA samples were stored at −20 °C until use.

### 2.3. Selection of Candidate Reference Genes and Design of RT-qPCR Primers

In total, 20 candidate genes (*TCTP*, *ACT7*, *GAPDH*, *UBC36*, *UBC7*, *EF2*, *CYP20-2*, *UBQ*, *TUA*, *UBC28*, *ICln*, *ubiquinone*, *PPR*, *SDE2*, *EIF4A-3*, *helicase-15*, *PAB2*, *CYP9*, *RHA2A*, and *EF1α*) were selected from the transcriptome database of *L. megaphylla* based on reference genes reported in the literature. All primers were designed using the qPCR primer quest tool (https://sg.idtdna.com/pages/tools/primerquest?returnurl=%2Fprimerquest%2FHome%2FIndex, accessed on 9 June 2022) based on the coding sequences (CDS) in the transcriptome database of *L. megaphylla* (Supplementary Table S1). Details of these candidate reference genes and primers are shown in Table 2.

### 2.4. RT-PCR and RT-qPCR Data Analysis

To verify the accuracy of the designed primers, each pair was used for RT-PCR amplification. Each 20 µL reaction system contained 2 µL of 10 µM forward and reverse primers, 1 µL cDNA, 10 µL 2 × Rapid Taq Master Mix (Vazyme, Nanjing, China) and 7 µL ddH_2_O. The reaction was carried out at 95 °C for 3 min, followed by 35 cycles of 95 °C for 30 s, 60 °C for 30 s and 72 °C for 15 s, with a final extension at 72 °C for 2 min. The PCR products were visualized by 1% (*w*/*v*) agarose gel electrophoresis.

To monitor the E-value, the cDNA templates from all samples were serially diluted five-fold (cDNA:water, v:v). RT-qPCR was performed for each pair of primers to obtain Ct values and to establish a standard curve; the R^2^, slope and E-values were calculated with Microsoft Office Excel 2019 using the following formula: E = (5^−1/slope^ − 1) × 100% [43].

All RT-qPCRs were performed with an Applied Biosystems^TM^ (ABI) QuantStudio^TM^ 5 real-time PCR system (ABI, Los Angeles, CA, USA) using the following amplification procedure: 95 °C for 30 s, followed by 40 cycles of 95 °C for 5 s and 60 °C for 30 s. A melting curve was generated at 60–95 °C. Each 20 µL RT-qPCR reaction (10 µL 2 × SYBR^®^ Green *Pro Taq* HS Premix, 2 µL 5-fold diluted cDNA, 0.4 µL 10 µM forward primer, 0.4 µL 10 µM reverse primer, 0.4 µL 4 µM ROX Reference Dye, and 6.8 µL RNase-free water) was prepared according to the instructions for SYBR^®^ Green Premix Pro Taq HS qPCR Kit (Rox Plus) (Accurate Biology, Shanghai, China). A negative control without the addition of cDNA was used to test for background amplification. Three technical replicates were performed for each sample, and the mean was used for RT-qPCR analysis.

### 2.5. Candidate Reference Gene Expression Stability Analysis

CT values were used to assess the expression levels of candidate reference genes in all samples by RT-qPCR. Four common algorithms, namely, delta Ct (ΔCt), geNorm (version 3.5), NormFinder (version 0.953) and BestKeeper (version 1.0), were used to evaluate the stability of the expression of the candidate reference genes in the different experimental groups. In geNorm and NormFinder, the M value reflects the stability of each candidate reference gene [28,29], with a smaller M value indicating higher stability. The geNorm package also determines the number of optimal reference genes based on the ratio V_n/n+1_ by calculating pairwise variations in the normalized factor after introducing a new internal reference gene [28]. A cut-off value of 0.15 was used for pairwise variation. If the value of V_n/n+1_ was less than 0.15, n was selected for the number of optimal internal reference genes; if the value of V_n/n+1_ was greater than 0.15, n + 1 was selected. For BestKeeper, the values of CV and SD were used to evaluate the relative expression stability of each candidate gene [29]. The smaller the CV and SD values are, the more stable the gene. Finally, the RefFinder program (http://blooge.cn/RefFinder/, accessed on 25 September, 2022) was used to comprehensively rank the candidate reference genes by ΔCt, geNorm, NormFinder, and BestKeeper as previously described [31].

### 2.6. Validation of Candidate Reference Genes by RT-qPCR

The NAC and ERF transcription factors function as central switches of growth, development and various abiotic/biotic stress responses in plants [44,45,46,47,48]. Hence, *LmNAC83* and *LmERF60* genes were selected as targets to determine the reliability of the most stable and unstable reference genes. The primers for these two genes are shown in Supplementary Table S2. The expression levels of these genes were calculated using the 2^−∆∆Ct^ method [35]. The RT-qPCR analysis was carried out using RNA from three biological replicates.

## 3. Results

### 3.1. Verification of Amplicon Size, Primers Specificity and PCR Amplification Efficiency

A total of 20 candidate genes from *L. megaphylla* were selected as potential reference genes for the normalization of target gene transcript levels using RT-qPCR. Standard PCR amplification with primers (Table 2, Supplementary Table S1) targeting the candidate genes was performed with reverse-transcribed cDNA from each sample as templates. Agarose gel electrophoresis indicated that all PCR products were single bands of the expected sizes, indicating that the primers were specific (Supplementary Figure S1). The melting curves, obtained after 40 cycles of amplification by RT-qPCR, showed single peaks, which also verified that the primers for the 20 candidate reference genes had strong specificity (Supplementary Figure S2). Standard curves, generated using a five-fold serial dilution for each candidate gene, had linear correlation coefficients (R^2^) greater than 0.99 for each specific primer pair, and the amplification efficiencies of the RT-qPCR reactions were 64.42–107.17% (Table 2). Because the amplification efficiencies of *ICIn* (64.42%), *UBQ* (85.95%), *SDE2* (86.75%), and *EF1α* (83.49%) were less than 90%, and the R^2^ values of *CYP95* and *RHA2A* were <0.99, these six candidate genes, based on the designed primers, were not appropriate for validating expression. Therefore, the remaining 14 candidate genes were used for subsequent experiments.

### 3.2. Transcript Abundance of Candidate Reference Genes

**Figure 1:**
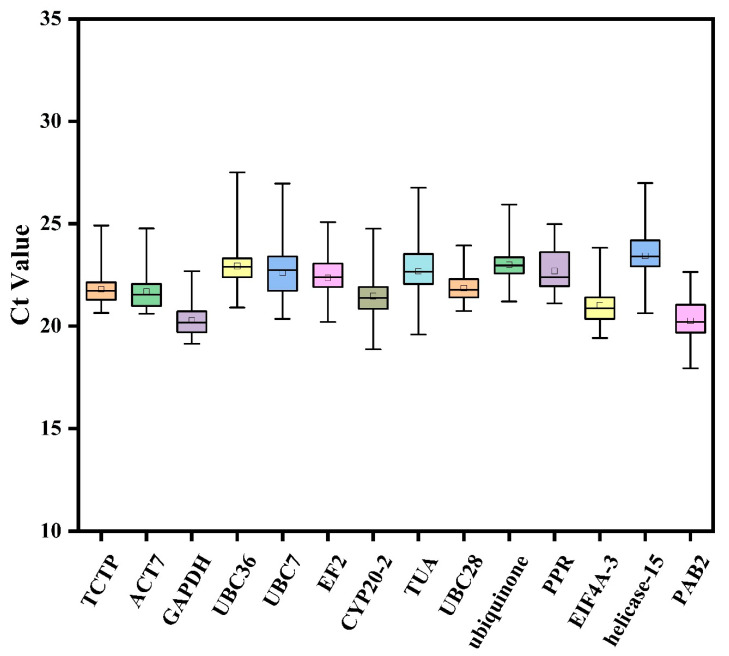
Distribution of average cycle threshold (Ct) values of the 14 candidate reference genes across all 38 samples from *L. megaphylla*. Boxes represent the interquartile range, with the middle solid lines indicating the median Ct values. The upper and lower boundaries of each box represent the 75th and 25th percentiles, respectively. The upper and lower bars represent the maximum and minimum Ct values, respectively, and the small squares represent the average values.

The transcript abundance of 14 candidate reference genes was estimated using the average cycle threshold (Ct) values for RNA extracted from different tissues or plants grown under different experimental conditions. All candidate reference genes were expressed at a wide range of transcript levels under the different experimental conditions. The gene expression level is negatively correlated with the Ct value, which means that a gene with a higher transcript level has a smaller Ct value. As shown in Figure 1, the minimum Ct value was 17.94, and the maximum was 27.51. Among the genes, *UBC36* exhibited the highest transcript abundance, with the minimum, median and maximum Ct values of 20.91, 22.90, and 27.51, respectively. *PAB2* showed the lowest expression abundance, with minimum, median and maximum Ct values being 17.94, 20.20, and 22.65, respectively. Compared with other genes, *GAPDH*, *UBC28,* and *TCTP* had obviously narrow range of Ct values (19.14–22.69, 20.74–23.95, and 21.10–24.98, respectively), indicating that they have a relatively stable expression level. While all the candidate reference genes exhibited significant expression abundance, none of the candidate genes were expressed stably in all samples. Thus, it is necessary to screen the appropriate internal reference genes for *L. megaphylla* under different experimental conditions or tissue types.

### 3.3. Estimation of the Stability of the Reference Genes under Different Experimental Conditions

To identify the optimal reference genes for the normalization of gene expression analysis in *L. megaphylla*, the stability of the 14 candidate genes was assessed by four different algorithms. The RefFinder software was used for overall ranking.

### 3.4. Delta Ct Method Analysis

**Figure 2:**
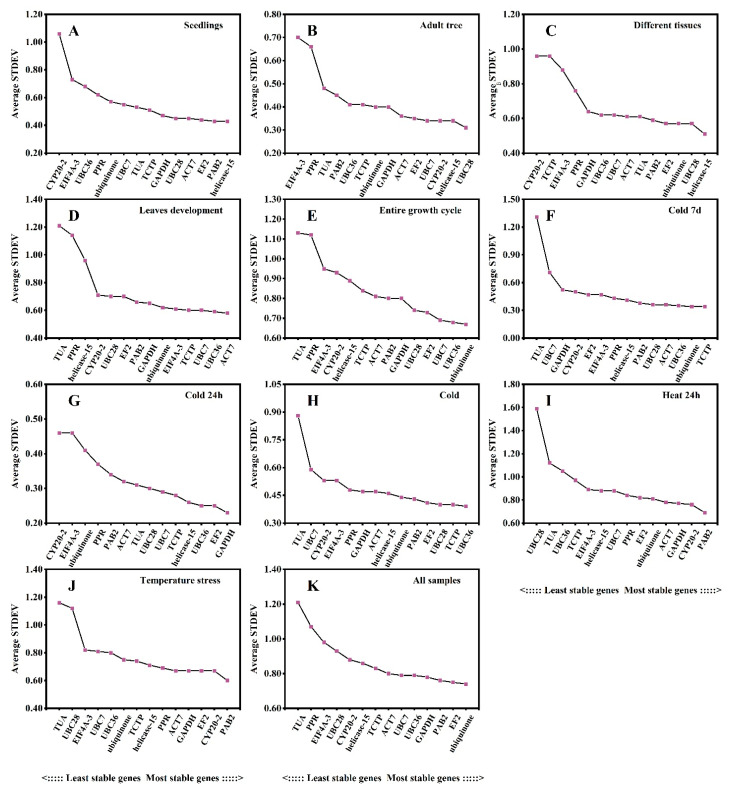
Ranking of expression stability of the 14 candidate reference genes in *L. megaphylla* using ΔCt analysis. Genes are listed across bottom of each plot in order of increasing stability from left to right. Results from (**A**) different seedling tissues; (**B**) different adult tree tissues; (**C**) all seedling and adult tree tissues; (**D**) 16 leaf developmental stages; (**E**) entire growth cycle including all different seedlings and adult tree tissues as well as 16 leaf developmental stages; (**F**) cold treatment for 7 days; (**G**) cold treatment for 24 h; (**H**) cold treatments including 7 days and 24 h; (**I**) heat treatment for 24 h; (**J**) different temperature treatments including cold and heat; (**K**) total samples.

The delta Ct (ΔCt) method ranks the stability of candidate reference genes based on the relative expression levels of “gene pairs” in each group of sample comparisons, while the mean standard deviation of gene expression differences (STDEV) is inversely proportional to its stability using the raw Ct value [34]. The stability of the transcript levels of each candidate reference gene was evaluated based on the STDEV value. The gene with the minimum STDEV value was regarded as the most stably expressed gene. The results demonstrated that the optimal reference genes were different in the different experimental sets (Figure 2). In seedling samples, *helicase-15* and *PAB2* showed the lowest ΔCt values (0.43), indicating the most stability (Figure 2A), while *UBC28* (0.31) was the most stably expressed gene in adult trees (Figure 2B). In other tissues, *helicase-15* (0.51) and *UBC28* (0.57) were the most stable reference genes (Figure 2C), which is consistent with the results for seedlings and adult trees. *ACT7* (0.58) and *UBC36* (0.59) were more stable across different leaf developmental stages (Figure 2D). Data analyses from the entire growth cycle indicated that *ubiquinone* (0.67) and *UBC36* (0.68) were the most stable (Figure 2E). *UBC36* showed good stability in all three cold stress sets (Figure 2F–H). *PAB2* (0.69) had the highest stability under heat treatment (Figure 2I). Across all temperature stresses, *PAB2* (0.6) was the most stably expressed gene (Figure 2J). For all samples, ubiquinone (0.74), *EF2* (0.75) and *PAB2* (0.76) showed the most stability (Figure 2K). *TUA* had relatively higher Ct values, indicating that it was the least stable reference gene in most of the experimental sets (Figure 2A–K).

### 3.5. geNorm Analysis

The stability of the reference genes was ranked by calculating the average expression stability values (M value) using the geNorm program, taking into account only similar intergroup variation [35].

**Figure 3:**
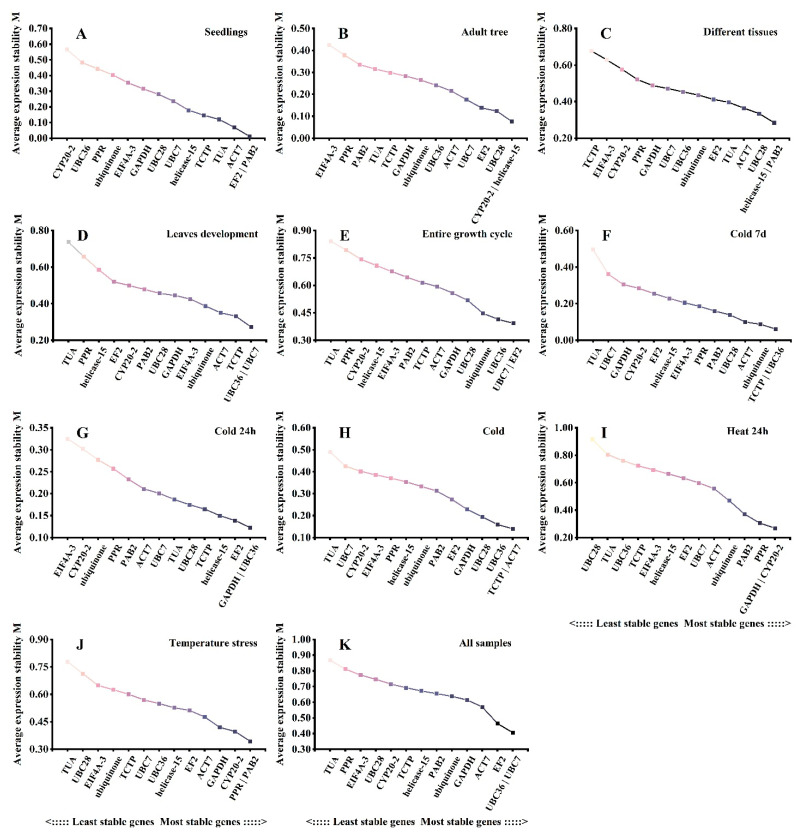
Ranking of expression stability of the 14 candidate reference genes in *L. megaphylla* using geNorm analysis. Results from (**A**) different seedling tissues; (**B**) different adult tree tissues; (**C**) all seedling and adult tree tissues; (**D**) 16 leaf developmental stages; (**E**) entire growth cycle including all different seedling and adult tree tissues as well as 16 leaf developmental stages; (**F**) cold treatment for 7 days; (**G**) cold treatment for 24 h; (**H**) cold treatments including 7 days and 24 h; (**I**) heat treatment for 24 h; (**J**) different temperature treatments including cold and heat; (**K**) total samples.

Genes with an M value less than 1.5 were considered stably expressed, with smaller M values indicating a more stable gene [28]. The geNorm analysis results for the 14 candidate genes in the different experimental sets are shown in Figure 3A–K. The M values of the 14 candidate reference genes were less than 1.5 under the different experimental conditions (Figure 3). *EF2* and *PAB2* had the highest stability in seedlings, with M values of 0.012 (Figure 3A), while *CYP26-2* (0.076) and *helicase-15* (0.076) were most stably expressed in the adult tree (Figure 3B). Between the different tissue sets, *PAB2* and *helicase15* were the optimal candidate genes, which is similar to the results in seedlings and adult trees (Figure 3C). The genes *PAB2* and *helicase-15* had the lowest stability values (0.285), which is consistent with the results of the ΔCt analysis. The two most stably expressed genes among the different leaf developmental stages were similar to those for the entire growth cycle, namely, *UBC36* and *UBC7* (Figure 3D,E). These results are also similar to those of the ΔCt analysis. *UBC36* was more stable than the other candidate reference genes under the cold treatments (Figure 3F–H) and was the same regardless of whether the cold treatment lasted for 7 days or 24 h. *GAPDH* (0.269), *CYP20-2* (0.269), and *PPR* (0.306) were more stable than the other candidate genes under heat treatment (Figure 3I). However, *PPR* and *PAB2* (0.343) exhibited the strongest stability under temperature stress (Figure 3J). These results are consistent with the results of the ΔCt analysis. *UBC36* and *UBC7* (0.406) showed the strongest stability in all samples (Figure 3K). In contrast, *TUA* and *PPR* were the least stable across most sets.

**Figure 4:**
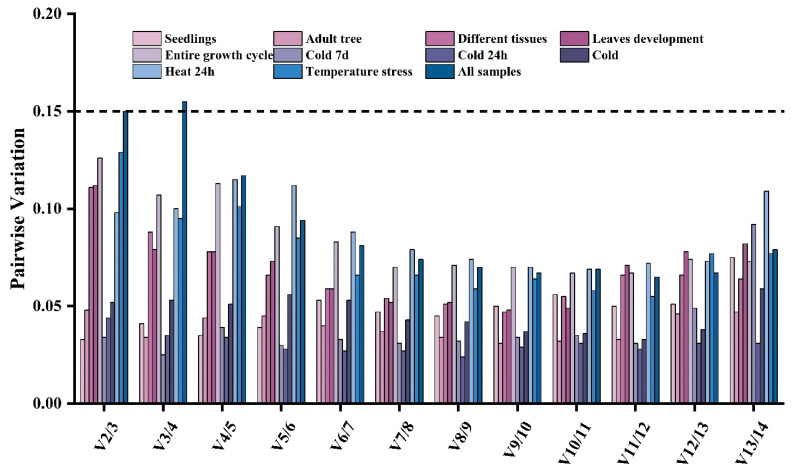
Pairwise variation values calculated by geNorm were used to determine the optimal number of reference genes from the 14 candidates in *L. megaphylla*. The cutoff value of V_n/n+1_ to determine the optimal number of reference genes for RT-qPCR normalization was 0.15.

Best practices include using multiple reference genes as internal controls for standardization to improve the accuracy of RT-qPCR data [28,49]. The number of optimal genes for standardization of the different datasets from *L. megaphylla* was calculated using the V_n/n+1_ function of geNorm, with a threshold of 0.15 (Figure 4). Interestingly, the values of V_2/3_ were less than 0.15 for most experimental groups (0.033, 0.045, 0.111, 0.112, 0.126, 0.034, 0.044, 0.052, 0.098, and 0.129) except for the ‘all samples’ group, as shown in Figure 4. This suggested that two was the optimal number of reference genes for each type of samples. However, for the ‘all samples’ set, the V_2/3_ and V_3/4_ values were greater than 0.15, and it is not until four reference genes are used (V_4/5_ value of 0.117) that the values is less than 0.15. Thus, at least four genes are required to obtain accurate results across many tissues and treatments.

### 3.6. NormFinder Analysis

**Figure 5:**
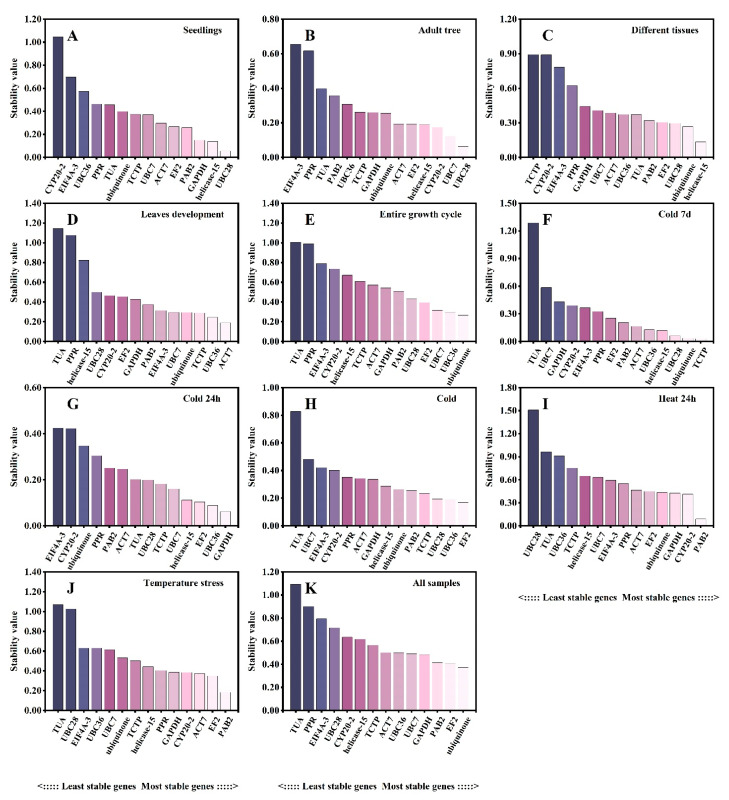
Ranking of expression stability of the 14 candidate reference genes in *L. megaphylla* using NormFinder analysis. Results from (**A**) different seedling tissues; (**B**) different adult tree tissues; (**C**) all seedling and adult tree tissues; (**D**) 16 leaf developmental stages; (**E**) entire growth cycle including all different seedling and adult tree tissues as well as 16 leaf developmental stages; (**F**) cold treatment for 7 days; (**G**) cold treatment for 24 h; (**H**) cold treatments including 7 days and 24 h; (**I**) heat treatment for 24 h; (**J**) different temperature treatments including cold and heat; (**K**) total samples.

To further determine the reliability of the results obtained by the geNorm algorithm, the NormFinder application was used to evaluate both the intra- and inter-group variation to calculate the stability of the candidate reference genes, with lower values of inter- and intra-group variation corresponding to increased stability of the candidate gene [48]. The results of the NormFinder analysis are shown in Figure 5. *UBC28* (0.055) and *helicase-15* (0.138) were the most stable genes in seedlings, while *UBC28* (0.063) and *UBC7* (0.123) were the most stable genes in adult trees (Figure 5A,B). The best combination of reference genes for different tissues was *helicase-15* (0.134) and *ubiquinone* (0.267) (Figure 5C). In the combined leaf developmental stages set, the optimal combination of candidate reference genes was *UBC7* (0.190) and *ACT7* (0.245), while over the entire growth cycle, the most stable candidate genes were *ubiqunone* (0.267) and *UBC36* (0.295) (Figure 5D,E). *TCTP* (0.030) and *ubiqunone* (0.035) were the most stable genes under cold treatment for 7 d, while *GAPDH* (0.062) and *UBC36* (0.088) were the most stable genes under cold treatment for 24 h (Figure 5F,G). Including both cold stress treatments, the candidate reference genes *EF2* (0.168) and *UBC36* (0.193) were the most stable (Figure 5H). Under heat treatment for 24 h, *PAB2* (0.089) and *CYP20-2* (0.414) were the most stable reference genes (Figure 5I). Under all temperature stresses, *PAB2* (0.185) and *EF2* (0.349) showed the highest stability (Figure 5J). The best combination of reference genes to compare all sample sets was *ubiquinone* (0.375), *EF2* (0.408), *PAB2* (0.413), and *GAPDH* (0.483) (Figure 5K). Notably, *TUA* and *PPR* were the least stable genes in most sets, similar to the results calculated by geNorm. In general, the results of the NormFinder analysis for the 14 candidate reference genes under different experimental conditions were similar to the results of the ΔCt and geNorm analyses.

### 3.7. BestKeeper Algorithm

The BestKeeper algorithm evaluates the most stable candidate reference genes based on the standard deviation (SD) and coefficient of variance (CV) of the average cycle threshold (Ct) values [16,19,32]. In general, the smaller the SD value is, the more stable the gene is. As shown in Table 3, the genes *UBC7* (0.06 ± 0.24) and *helicase-15* (0.14 ± 0.57) had the lowest SD values in seedlings, while *GAPDH* (0.09 ± 0.48) and *TCTP* (0.18 ± 0.83) were the most stable genes in adult trees. The genes with the most stable expression across different tissues were *ACT7* (0.29 ± 1.36) and *UBC28* (0.31 ± 1.40), while the expression of *UBC28* (0.60 ± 2.76) and *GAPDH* (0.66 ± 3.24) was the most stable across different leaf developmental stages. The genes with the most stable expression across the entire growth cycle were *UBC28* (0.58 ± 2.64) and *TCTP* (0.59 ± 2.73). Under cold treatment, *PPR* (0.18 ± 0.82) and *EIF4A-3* (0.19 ± 0.91) were the most stable genes, while under heat treatment, *UBC28* (0.22 ± 1.03) and *ubiquinone* (0.54 ± 2.35) were the most stable genes. Under both types of temperature stress, the expression of *UBC28* (0.37 ± 1.70) and *PPR* (0.42 ± 1.86) were the most stable. For all samples, the top four most stable reference genes were *UBC28* (0.51 ± 2.32), *TCTP* (0.56 ± 2.57), *GAPDH* (0.60 ± 2.98) and *ubiquinone* (0.64 ± 2.76), while *TUA* showed the highest value and thus the lowest expression stability.

### 3.8. Comprehensive Stability Analysis Using RefFinder

**Figure 6:**
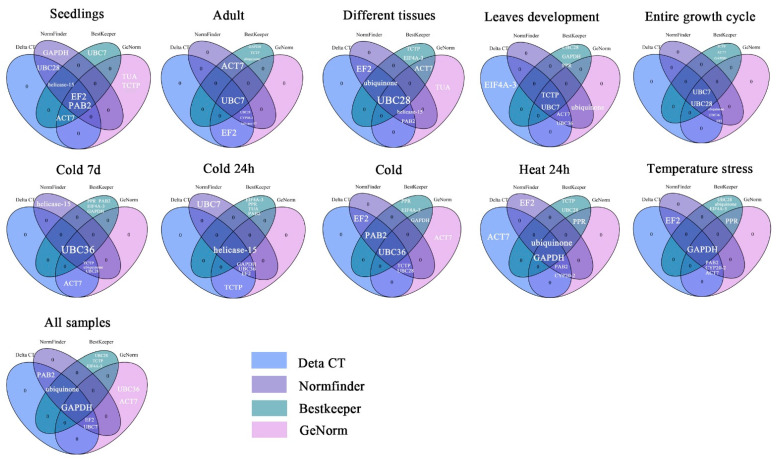
Venn diagrams displaying the overlap between the top five most stable reference genes as ranked by ΔCt, geNorm, NormFinder, and BestKeeper for the different datasets. Genes in the overlapping area were confirmed by more than one algorithm. The light purple, purple, light green and pink ellipses contain the top five most stable reference genes selected by ΔCt, geNorm, NormFinder, and BestKeeper, respectively.

Since the different algorithms determined different stability rankings for the candidate genes, the program RefFinder was used to calculate the geometric mean of the ranking results from the four methods [16]. The comprehensively ranked candidate genes (Table 4) did not present one or two universal reference genes for the normalization of gene expression data for all samples. However, the consensus for the top two genes in adult trees during leaf development, under cold treatment for 7 days or 24 h, and under heat treatment for 24 h was consistent with the results of the ΔCt and NormFinder analyses. The top two reference genes in seedlings or under temperature stress were consistent with the results of the ΔCt or geNorm analysis, respectively. In the other experimental conditions, the top two most stable genes in the overall ranking appeared in either the top two or top three positions in one of the other four algorithms. In addition, all analyses revealed that *TUA* was the most unstable gene. We then analyzed the top five most stable genes as determined using the ΔCt, geNorm, NormFinder, BestKeeper, and RefFinder algorithms (Figure 6). The top two most stable reference genes selected using RefFinder for the various experimental sets were ranked highly by three or four of the other software programs.

### 3.9. Validation of Reference Genes

**Figure 7:**
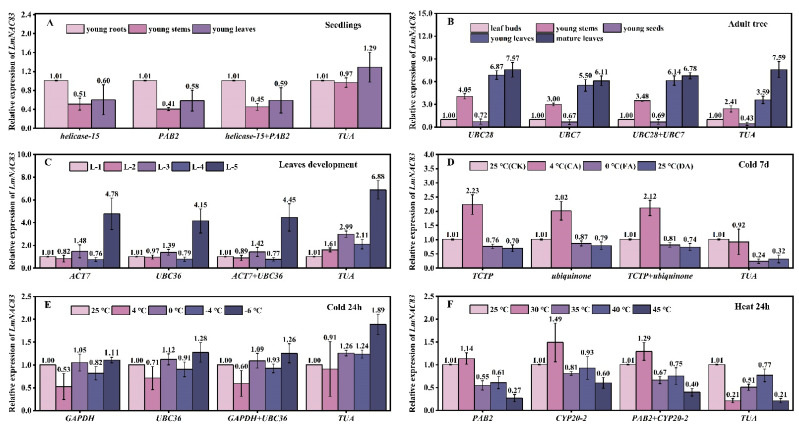
Relative expression of *LmNAC83* in six experimental sample sets. The results were normalized using the stable reference genes (alone or in combination) or an unstable gene in six sample sets, including (**A**) different seedling tissues; (**B**) different adult tree tissues; (**C**) different leaf developmental stages; (**D**) cold treatment for 7 days; (**E**) cold treatment for 24 h; and (**F**) heat treatment for 24 h. The bars indicate the standard deviation (±SD) evaluated from three biological replicates.

**Figure 8:**
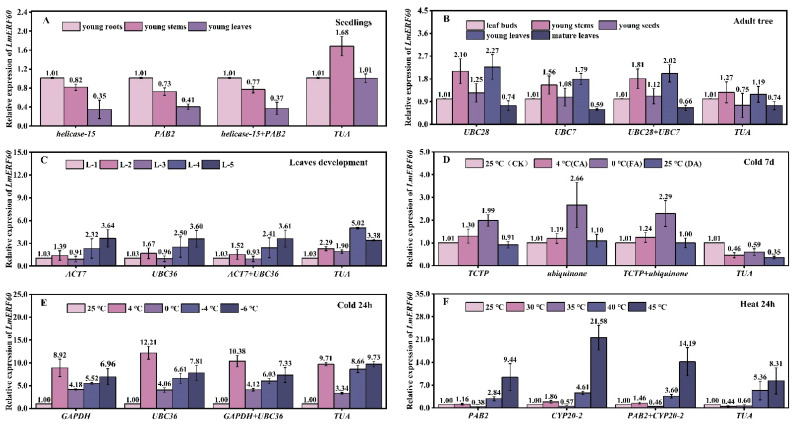
Relative expression of *LmERF60* in six experimental sample sets. The results were normalized using the stable reference genes (alone or in combination) or an unstable gene in six sample sets, including (**A**) different seedling tissues; (**B**) different adult tree tissues; (**C**) different leaf developmental stages; (**D**) cold treatment for 7 days; (**E**) cold treatment for 24 h; and (**F**) heat treatment for 24 h. The bars indicate the standard deviation (±SD) evaluated from three biological replicates.

To verify the reliability of the selected reference genes, the transcripts of two genes were quantified using either different combinations of the two most stable genes, using a single reference gene or using the relatively unstable reference gene *TUA* under six different experimental conditions. The test genes were *NAC* and *ERF*, both of which show relatively high abundance levels, with fragments per kilobase of transcript per million fragments mapped (FPKM) values of approximately 22–223. The relative expression patterns and levels of the *LmNAC83* gene showed similar trends when the most stable genes were used alone or in combination as reference genes for standardization (Figure 7A–F). In contrast, the relative expression levels of *LmNAC83* showed significant fluctuations when the relatively unstable gene *TUA* was used for relative quantification. For example, under heat treatment for 24 h, the expression levels of *LmNAC83* in leaves was the highest at 30 °C when using the most stable genes as the reference genes. However, the relative expression of *LmNAC83* was low when using the unstable gene (*TUA*) as the reference gene (Figure 7F). In addition, the expression levels and trends of the *LmERF60* gene (Figure 8) were very similar to those found in the analysis of *LmNAC83*. It is evident that the use of unstable internal reference genes for gene expression analysis in *L. megaphylla* can lead to unreliable results. This test illustrates the importance of screening stable internal reference genes under different experimental conditions.

## 4. Discussion

Changes in plant secondary metabolism are closely related to the transcriptional activities of key genes, and gene expression analysis is a key technique for understanding the mechanisms involved in these processes [32,49]. RT-qPCR is the most accurate technique to obtain gene expression profiles that relate to biological function and regulatory networks [50,51,52]. However, the accuracy of RT-qPCR results mainly depends on using optimal internal reference genes that are stably expressed in samples across different experimental conditions. Reference genes are crucial for normalization of gene expression data and avoiding experimental errors by minimizing non-biological variation between different samples [24,33,35]. To ensure the accuracy of experiments, it is important to select suitable reference genes for each species that similar transcript levels under different experimental conditions.

*Lindera megaphylla* is an ecologically important and dominant broad-leaved evergreen tree species that is naturally distributed in the warm-temperate and subtropical zones of China [1]. This tree contributes to the seasonal landscape and produces volatile compounds with strong effects on bacteria and toxic gases. For example, the terpenes produced have a strong antibacterial effect [6,10,53]. *L. megaphylla* is also used as a medicinal plant [2]. However, few studies have focused on the molecular biology of *L. megaphylla* due to limited genomic information, and to date, no reference genes have been reported. In this study, we obtained transcriptome databases of different tissues of *L. megaphylla* and identified appropriate internal reference genes for use when studying the expression of genes. We initially used 40 internal reference genes from published model plants such as *Arabidopsis thaliana* and other species in the Lauraceae family to screen for constitutively expressed reference genes from the transcriptome database of *L. megaphylla*. We obtained 20 candidate genes based on an FPKM value > 50. As shown in Table 2, the final 14 candidate genes were selected based on their E-values in the range of 91.04–107.17% and R2 values in the range of 0.991–0.999, which indicated that the primer pairs for standardized evaluation by RT-qPCR had high sensitivity and accuracy. In addition, the average Ct values of the candidate genes ranged from 17.94 (*PAB2*) to 27.51 (*UBC36*), indicating different expression levels (Figure 1). The results obtained are similar to those of many previous studies, such as on *Cryptomeria fortune* [33], *Gerbera hybrid* [19], and *Piper species* [48]. The results indicate that none of the reference genes had constant expression levels under all tested experimental conditions or in different species. Thus, it is necessary to carefully select the most appropriate reference gene to ensure the accuracy of RT-qPCR analysis for specific conditions and with specific materials. In this study, we combined four statistical algorithms (ΔCt, geNorm, NormFinder, and BestKeeper) to assess the expression stability of 14 candidate genes (*TUA*, *PPR*, *EIF4A-3*, *CYP26-2*, *helicase-15*, *TCTP*, *ACT7*, *PAB2*, *GAPDH*, *UBC28*, *EF2*, *UBC7*, *UBC36,* and *ubiquinone*) in different tissues across 16 different leaf developmental stages, and under different temperature stresses. The results demonstrated that the optimal reference genes were not the same under different conditions (Table 4).

There were slight differences in the rankings of candidate reference genes between the different algorithms. However, analysis by the ΔCt and NormFinder algorithms consistently identified the most stable or unstable candidate reference genes for most experimental sets, while in a few experimental groups, the expression patterns of similar genes were the most or least stable in geNorm and NormFinder. The ranking of candidate genes by BestKeeper suggested some differences compared to the other algorithms. For example, for different leaf developmental stages, the ΔCt and NormFinder platforms indicated that the *ACT7* and *UBC36* genes were the most stable. geNorm placed these genes in fourth and first place, while the BestKeeper program placed these genes in sixth and seventh place. Although the rankings of candidate genes produced by the different algorithms were slightly different, the top five stable candidate genes selected by the algorithms were similar for each group of experimental conditions (Table 3). For instance, *ACT7*, *UBC36*, *TCTP*, *UBC7,* and *ubiquinone* were the top five most stable genes based on the geNorm and NormFinder analyses across the leaf development stages, and the ΔCt analysis showed similar results, except for *ubiquinone*. BestKeeper analysis identified two stable genes: *UBC7* and *TCTP*. Numerous other studies have found similar differences between the outputs of geNorm and NormFinder [32,54], and many studies also demonstrated that these subtle differences result from the use of different algorithm models [33,34,55].

To comprehensively synthesize the results of the four algorithms, RefFinder was utilized to rank the identified candidate genes in *L. megaphylla*. This analysis plays an important role in integrating the screening results of reference genes from other algorithms by assigning an appropriate weight to each gene and calculating the geometric mean of its weights to produce a final ranking [32,34,35]. Fortunately, we found that the results from RefFinder were similar to those of the different algorithms in each experimental set, proving that RefFinder can assess and screen the optimal reference genes [56], as shown in Figure 6 and Table 4.

The results also indicate that we screened and identified the optimal reference gene combinations for use in *L. megaphylla* samples generated under different experimental conditions. To compare expression in different seedling tissues, *helicase-15* and *PAB2* were the most suitable, whereas *UBC28* and *UBC7* were most stably expressed in different adult trees tissues. When two different groups of tissues were analyzed, *helicase-15* and *UBC28* emerged as the most stable gene combination. For different leaf developmental stages, *ACT7* and *UBC36* were best, whereas *ubiquinone* and *UBC7* were best when analyzing samples over the entire growth cycle. Interestingly, for cold stress of 7 days and 24 h, the optimal reference genes were *TCTP* + *ubiquinone* and *GAPDH* + *UBC36*, respectively. For heat treatment, the best reference genes were *PAB2* and *CYP20-2*, while for overall temperature stress, *PAB2* and *PPR* were most stable. When all samples were tested, *ubiquinone*, *EF2*, *UBC7,* and *GAPDH* were the optimal candidate reference genes overall for the normalization of gene expression in *L. megaphylla* (Table 4). These analyses are sufficient to demonstrate the necessity of screening suitable internal reference genes under different experimental conditions for each species.

With increasing demand for accurate scientific data, it has become important to screen for the best internal reference genes in a greater number of plant species and for different experimental treatments [19,32,49]. Some housekeeping genes involved in cytoskeleton structure or primary metabolism, including *ACT*, *TUA,* and *EIF4α*, are extensively used as reference genes in many plant species. For example, *ACT2/7* and *TUA* are the three most stable genes across different developmental stages of *Glycine max* [57]. *AhyACT*, *AhyMDH*, and *AhyEF-1a* are the most stable genes in different tissues of amaranth [56]. Research on bamboo revealed that *eIF4α* was most stable in different organs, while *CYP*, *eEF1α,* and *UBQ5* were found to be the optimal reference genes for different developmental stages of *Bambusa tulda* [18]. In *Litsea cubeba*, *F-BOX*, *EF1α,* and *EIF4α* were the most stable reference genes across different tissues and developmental stages [40]. In the present study, *TUA* was the least stable candidate gene in *L. megaphylla,* as calculated by the different programs, while the best combinations of genes were *helicase-15* + *UBC28* and *ACT7* + *UBC36* in different tissues and developmental stages.

Zhong et al. [24] found that *ACT* was stably expressed in high-temperature-stressed *Psoralea corylifolia*. Chen et al. [22] indicated that *EF-1α* was the most stably expressed and suitable reference gene under heat and cold treatments. In this study, *PAB2* + *CYP20-2* and *UBC36* +*TCTP* were identified as the most stable reference genes under heat and cold treatments, respectively. The experimental results once again show the importance of screening the best reference genes under different experimental conditions for different species.

## 5. Conclusions

*helicase-15* and *UBC28* can be used as internal reference genes when detecting gene expression pattern in different tissues of *L. megaphylla*. *ACT7* and *UBC36* were considered to be the most suitable internal reference genes at different leaf developmental stages of *L. megaphylla*. *UBC36*, *TCTP*, *PAB2,* and *CYP20* were optimum reference genes when *L. megaphylla* was suffering from abiotic stress. Among them, *UBC36* and *TCTP* were the best under cold treatment, while *PAB2* and *CYP20-2* were the best under heat treatment. We recommend that two internal reference genes be used to normalize RT-qPCR data in future experiments. In short, our results lay a foundation for future molecular biology research on *L. megaphylla*.

## Figures and Tables

**Table 1:** Six experimental sets of *L. megaphylla*.

Experimental Sample Sets	Tissue Type	Biological Replicates	Sampling Dates	Total Number of Samples
Different tissues of oneyear-old seedlings	Roots, stems and leaves	3	1	3
Different tissues of adult trees	Leaf buds, young stems, young seeds, young leaves and mature leaves	3	1	15
Developmental stages	Leaves	3	15	45
Cold stress for 7 days	Leaves	3	4	12
Cold stress for 24 h	Leaves	3	5	15
Heat stress for 24 h	Leaves	3	5	15

**Table 2:** Candidate reference genes and designed primers for RT-qPCR normalization in *L. megaphylla*.

Gene Symbol	Gene Name	Primer Sequence (5′ → 3′)	Product Length (bp)	Standard Curve	E (%)	R²
*TCTP*	translationally controlled tumor protein	F:GTTTCTCACCCTCCAACTTAGG R:CATTTCGCCTCCAGGAACA	102	y = −2.4502x + 29.195	95.070	0.9992
*ACT7*	actin-related protein 7	F:AAGCCAACAGGGAGAAGATG R:CACCCGAGTCCAGAACAATAC	132	y = −2.3523x + 28.25	103.003	0.9971
*GAPDH*	Glyceraldehyde 3-phosphate dehydrogenase	F:CGGAGGATGATGTGGTTTCTAC R:GCGACAAGCTTGACAAAGTG	106	y = −2.3607x + 27.623	98.050	0.9994
*UBC36*	ubiquitin-conjugating enzyme E2 36	F:CCCGAAGGTTCGATTTCTCA R:TGAAGAGCAGGACTCCATTTATC	102	y = −2.3618x + 29.443	101.509	0.9978
*UBC7*	ubiquitin-conjugating enzyme E2 7	F:TCATGAGCTTCCCAGCAAATTA R:CGTCCGTCGGGATAAACATTAG	91	y = −2.442x + 29.477	96.932	0.9974
*EF2*	elongation factor 2-like	F:GCGGATAAGGGTAGGTTCTTT R:TTCTGGCCAGGAACATAGTTAG	104	y = −1.9725x + 27.627	96.045	0.9919
*CYP20-2*	peptidyl-prolyl cis-trans isomerase CYP20-2, chloroplastic	F:AACACCAACGGTAGCCAAT R:TCCAGAACCTGCCCAAATAC	86	y = −2.4129x + 27.719	101.307	0.9963
*UBQ*	polyubiquitin	F:CCTCGCCGACTACAATATTCA R:CACCTCCAGAGTAATCGTCTTC	115	y = −2.2086x + 23.796	85.947	0.9989
*TUA*	Alpha-tubulin	F:GCCTTACAACAGTGTGCTTTC R:ATCTAGAGATCGACGGCAGATA	106	y = −2.3673x + 27.37	101.414	0.9972
*UBC28*	ubiquitin-conjugating enzyme E2 28-like	F:ACAATTATGGGACCAGCAGATAG R:GGGTGGCTTGAATGGGTAAT	90	y = −2.3925x + 28.93	101.491	0.9972
*ICln*	chloride conductance regulatory protein ICln	F:TGAGCGACACCGATAGAGAA R:TAAATGCAAGGAGAGGCGTAAG	103	y = −2.6401x + 31.012	64.420	0.9953
*ubiquinone*	NADH dehydrogenase	F:ATCCGACGGGCGATTAAAG R:TCTAGCCTCTTCTTCCAGATACT	123	y = −2.1552x + 28.534	107.169	0.9975
*PPR*	pentatricopeptide repeatcontaining protein	F:CTTTAAGCCAGACCAGCAAATG R:TCCTCTTTCAGCCATCTTTCC	106	y = −2.3288x + 29.75	102.616	0.9976
*SDE2*	replication stress response regulator SDE2-like	F:TAGACGGGCGGACCAGAT R:GAGGAGGACGGTGCAGGAG	197	y = −2.7496x + 30.276	86.753	0.9912
*EIF4A-3*	eukaryotic initiation factor 4A-3like	F:TCTTTGTTGCGGTTGAGCG R:ACCAATCCACCTTTCTTTTCG	117	y = −2.8406x + 28.345	95.752	0.9918
*helicase-15*	DEAD-box ATP-dependent RNA helicase 15	F:CCTGGGAGAATACTGGCACTG R:GGCCTCGTCGTCCACATAA	249	y = −3.1364x + 30.269	92.361	0.9992
*PAB2*	polyadenylate-binding protein 2like	F:CCCAAGCTGTTGAGGATCTTA R:CCTTTCAGCTCCATCTCTCTTT	100	y = −2.4748x + 31.269	91.035	0.9919
*CYP95*	peptidyl-prolyl cis-trans isomerase CYP95 like	F:GGGTTCAGTCATCGTTACTCTT R:GCGTTCACTTCTTCCTCCATA	99	y = −2.4245x + 29.937	103.599	0.9898
*RHA2A*	E3 ubiquitin-protein ligase RHA2A	F:CTTTAGCGGGAGCGATGT R:CAAGCACTCTCTGTGGAAGA	112	y = −2.3815x + 31.29	93.904	0.9870
*EF1α*	Translation elongation factor EF1A	F:AAATGAGGAGGAGCGTGTAAAG R:CGCTGATCATGTTAGGGACATAG	128	y = −2.6481x + 30.807	83.491	0.8779

**Table 3:** Ranking of expression stability of the 14 candidate reference genes in *L. megaphylla* using BestKeeper analysis.

Ranking	Seedlings			Adult Tree			Different Tissues			Leaf Development			Entire Growth Cycle			Cold 7 d		
	Gene Name	SD	CV (%)	Gene Name	SD	CV (%)	Gene Name	SD	CV (%)	Gene Name	SD	CV (%)	Gene Name	SD	CV (%)	Gene Name	SD	CV (%)
1	*UBC7*	0.06	0.24	*GAPDH*	0.09	0.48	*ACT7*	0.29	1.36	*UBC28*	0.60	2.76	*UBC28*	0.58	2.64	*PPR*	0.06	0.27
2	*helicase-15*	0.14	0.57	*TCTP*	0.18	0.83	*UBC28*	0.31	1.40	*GAPDH*	0.66	3.24	*TCTP*	0.59	2.73	*PAB2*	0.11	0.55
3	*EF2*	0.22	0.93	*ubiquinone*	0.21	0.91	*TCTP*	0.37	1.71	*TCTP*	0.71	3.27	*UBC7*	0.69	3.01	*EIF4A-3*	0.14	0.68
4	*PAB2*	0.22	1.01	*UBC7*	0.24	1.08	*EIF4α*	0.37	1.80	*UBC7*	0.73	3.21	*ACT7*	0.69	3.20	*GAPDH*	0.21	1.04
5	*ACT7*	0.27	1.28	*ACT7*	0.25	1.20	*ubiquinone*	0.39	1.69	*PPR*	0.80	3.56	*GAPDH*	0.69	3.40	*UBC36*	0.23	1.01
6	*GAPDH*	0.28	1.32	*EIF4A-3*	0.27	1.30	*PTB*	0.39	1.85	*ACT7*	0.80	3.67	*ubiquinone*	0.75	3.27	*TCTP*	0.25	1.15
7	*UBC28*	0.3	1.35	*UBC28*	0.30	1.37	*TUA*	0.45	1.97	*EIF4A-3*	0.88	4.11	*UBC36*	0.78	3.44	*UBC28*	0.25	1.15
8	*PPR*	0.34	1.38	*TUA*	0.35	1.54	*helicase-15*	0.46	1.93	*UBC36*	0.89	3.97	*EIF4A-3*	0.78	3.69	*ACT7*	0.25	1.19
9	*TUA*	0.35	1.51	*UBC36*	0.36	1.58	*UBC36*	0.50	2.17	*CYP20-2*	0.89	4.21	*EF2*	0.89	4.04	*ubiquinone*	0.27	1.16
10	*TCTP*	0.37	1.73	*EF2*	0.36	1.64	*EF2*	0.60	2.66	*ubiquinone*	0.91	3.98	*PPR*	0.95	4.17	*CYP20-2*	0.32	1.47
11	*ubiquinone*	0.46	1.93	*CYP20-2*	0.4	1.85	*PPR*	0.69	2.92	*EF2*	1.05	4.78	*CYP20-2*	0.97	4.51	*helicase-15*	0.34	1.45
12	*EIF4A-3*	0.54	2.63	*helicase-15*	0.42	1.79	*UBC7*	0.69	2.97	*PAB2*	1.06	5.34	*PAB2*	1.02	5.05	*EF2*	0.40	1.80
13	*UBC36*	0.58	2.46	*PAB2*	0.43	2.05	*GAPDH*	0.73	3.62	*helicase-15*	1.38	6.02	*helicase-15*	1.14	4.91	*UBC7*	0.63	2.74
14	*CYP20-2*	0.80	3.40	*PPR*	0.61	2.65	*CYP26-2*	0.96	4.33	*TUA*	1.59	7.25	*TUA*	1.32	5.93	*TUA*	1.10	4.79
**Ranking**	**Cold 24 h**			**Cold**			**Heat 24 h**			**Stress Treatment**			**All Samples**					
	**Gene Name**	**SD**	**CV (%)**	**Gene Name**	**SD**	**CV** **(%)**	**Gene Name**	**SD**	**CV** **(%)**	**Gene Name**	**SD**	**CV** **(%)**	**Gene Name**	**SD**	**CV** **(%)**			
1	*EIF4A-3*	0.1	0.46	*PPR*	0.18	0.82	*UBC28*	0.22	1.03	*UBC28*	0.37	1.7	*UBC28*	0.51	2.32			
2	*PPR*	0.14	0.65	*EIF4A-3*	0.19	0.91	*ubiquinone*	0.54	2.35	*PPR*	0.42	1.86	*TCTP*	0.56	2.57			
3	*helicase-15*	0.3	1.29	*PAB2*	0.29	1.42	*TCTP*	0.77	3.5	*ubiquinone*	0.45	1.94	*GAPDH*	0.6	2.98			
4	*TUA*	0.32	1.41	*UBC36*	0.3	1.29	*PPR*	0.8	3.5	*EIF4A-3*	0.47	2.24	*ubiquinone*	0.64	2.76			
5	*PAB2*	0.33	1.64	*GAPDH*	0.32	1.55	*GAPDH*	0.82	3.98	*GAPDH*	0.49	2.39	*EIF4A-3*	0.66	3.14			
6	*GAPDH*	0.35	1.69	*ubiquinone*	0.34	1.45	*EF2*	0.83	3.55	*TCTP*	0.51	2.35	*ACT7*	0.68	3.12			
7	*UBC7*	0.36	1.54	*UBC28*	0.36	1.65	*PAB2*	0.91	4.39	*PAB2*	0.51	2.52	*UBC7*	0.71	3.08			
8	*ubiquinone*	0.37	1.59	*TCTP*	0.37	1.71	*EIF4A-3*	0.91	4.44	*CYP20-2*	0.63	2.96	*UBC36*	0.72	3.16			
9	*UBC36*	0.38	1.65	*helicase-15*	0.39	1.67	*CYP20-2*	0.95	4.38	*ACT7*	0.66	3.04	*EF2*	0.81	3.62			
10	*UBC28*	0.42	1.87	*ACT7*	0.4	1.83	*ACT7*	1.03	4.63	*EF2*	0.68	2.99	*PAB2*	0.82	4.05			
11	*EF2*	0.43	1.94	*EF2*	0.42	1.86	*UBC7*	1.09	4.5	*UBC7*	0.7	2.97	*PPR*	0.83	3.64			
12	*TCTP*	0.51	2.3	*UBC7*	0.43	1.84	*helicase-15*	1.17	4.81	*UBC36*	0.72	3.09	*CYP20-2*	0.85	3.94			
13	*ACT7*	0.53	2.43	*CYP20-2*	0.53	2.48	*TUA*	1.3	5.33	*helicase-15*	0.73	3.08	*helicase-15*	0.98	4.18			
14	*CYP20-2*	0.66	3.11	*TUA*	0.67	2.93	*UBC36*	1.36	5.64	*TUA*	1.21	5.2	*TUA*	1.22	5.4			

**Table 4:** Ranking of expression stability of the 14 candidate reference genes in *L. megaphylla* using RefFinder analysis.

Ranking	Seedlings		Adult Tree		Different Tissues		Leaf Development		Entire Growth Cycle		Cold 7 d	
	Gene Name	Geomean of Ranking Values	Gene Name	Geomean of Ranking Values	Gene Name	Geomean of Ranking Values	Gene Name	Geomean of Ranking Values	Gene Name	Geomean of Ranking Values	Gene Name	Geomean of Ranking Values
1	*PAB2*	2.21	*UBC28*	2.21	*helicase-15*	1.68	*ACT7*	2.11	*ubiquinone*	2.21	*TCTP*	1.86
2	*helicase-15*	2.21	*UBC7*	2.21	*UBC28*	2.91	*UBC36*	2.45	*UBC7*	2.45	*ubiquinone*	2.71
3	*EF2*	2.78	*CYP20-2*	2.78	*PAB2*	3.34	*UBC7*	2.99	*UBC36*	3.13	*UBC36*	2.94
4	*UBC28*	4.09	*helicase-15*	4.09	*ACT7*	3.72	*TCTP*	3.00	*UBC28*	3.34	*UBC28*	4.53
5	*ACT7*	4.36	*GAPDH*	4.36	*ubiquinone*	3.98	*GAPDH*	5.29	*EF2*	3.46	*PPR*	4.74
6	*UBC7*	4.58	*ACT7*	4.58	*EF2*	4.68	*UBC28*	5.30	*GAPDH*	5.45	*PAB2*	4.74
7	*GAPDH*	5.58	*ubiquinone*	5.58	*TUA*	6.19	*ubiquinone*	5.89	*TCTP*	6.00	*ACT7*	5.57
8	*TCTP*	7.27	*EF2*	7.27	*UBC36*	7.97	*EIF4A-3*	5.96	*ACT7*	6.88	*EIF4A-3*	7.00
9	*TUA*	7.33	*TCTP*	7.33	*EIF4A-3*	9.30	*PAB2*	8.82	*PAB2*	7.90	*helicase-15*	7.26
10	*ubiquinone*	10.22	*UBC36*	10.22	*UBC7*	9.46	*CYP20-2*	9.69	*EIF4A-3*	10.02	*GAPDH*	9.12
11	*PPR*	10.38	*TUA*	10.38	*TCTP*	9.53	*EF2*	10.22	*helicase-15*	10.94	*EF2*	9.64
12	*EIF4A-3*	11.93	*EIF4A-3*	11.93	*GAPDH*	10.68	*PPR*	10.72	*CYP20-2*	11.24	*CYP20-2*	10.74
13	*UBC36*	12.49	*PAB2*	12.49	*PPR*	11.24	*helicase-15*	12.24	*PPR*	12.17	*UBC7*	13.00
14	*CYP20-2*	14.00	*PPR*	14.00	*CYP20-2*	12.98	*TUA*	14.00	*TUA*	14.00	*TUA*	14.00
**Ranking**	**Cold 24 h**		**Cold**		**Heat 24 h**		**Stress Treatment**		**All Samples**			
	**Gene Name**	**Geomean of** **Ranking Values**	**Gene Name**	**Geomean of** **Ranking Values**	**Gene Name**	**Geomean of** **Ranking Values**	**Gene Name**	**Geomean of** **Ranking Values**	**Gene Name**	**Geomean of** **Ranking Values**		
1	*GAPDH*	1.57	*UBC36*	2.21	*PAB2*	2.30	*PAB2*	1.57	*ubiquinone*	2.21		
2	*UBC36*	2.45	*TCTP*	3.13	*CYP20-2*	2.45	*PPR*	2.91	*EF2*	3.22		
3	*helicase-15*	3.72	*UBC28*	3.60	*GAPDH*	2.59	*ACT7*	4.05	*UBC7*	3.64		
4	*EF2*	4.15	*EF2*	4.03	*ubiquinone*	3.76	*GAPDH*	4.16	*GAPDH*	3.94		
5	*UBC7*	6.40	*PAB2*	4.79	*PPR*	4.92	*EF2*	4.68	*UBC36*	4.12		
6	*TUA*	6.51	*ACT7*	5.33	*ACT7*	6.16	*CYP20-2*	4.68	*PAB2*	5.01		
7	*TCTP*	6.51	*PPR*	5.62	*EF2*	6.16	*UBC28*	6.85	*TCTP*	5.83		
8	*PPR*	7.18	*GAPDH*	6.32	*UBC28*	7.24	*ubiquinone*	7.19	*ACT7*	5.86		
9	*EIF4A-3*	7.24	*ubiquinone*	6.45	*TCTP*	7.95	*helicase-15*	8.17	*UBC28*	6.04		
10	*UBC28*	7.36	*EIF4A-3*	7.50	*UBC7*	8.89	*TCTP*	8.18	*helicase-15*	9.58		
11	*PAB2*	8.41	*helicase-15*	7.94	*EIF4A-3*	8.94	*EIF4A-3*	8.92	*EIF4A-3*	9.64		
12	*ACT7*	9.87	*CYP20-2*	11.72	*helicase-15*	9.64	*UBC7*	10.22	*CYP20-2*	10.47		
13	*ubiquinone*	10.84	*UBC7*	12.74	*UBC36*	12.47	*UBC36*	10.36	*PPR*	12.47		
14	*CYP20-2*	13.24	*TUA*	14.00	*TUA*	13.00	*TUA*	14.00	*TUA*	14.00		

## Data Availability

All data needed to evaluate the conclusions in this paper are present in the paper and/or the Supplementary Materials.

## Supplementary Materials

The following supporting information can be downloaded at: https://www.mdpi.com/article/10.3390/plants12112185/s1, Figure S1: The PCR products electrophoresis result of the 20 candidate reference genes; Figure S2: Melting curves of the 20 candidate reference genes in *L. megaphylla*. Table S1: The cDNA sequences of the 20 candidate reference genes from *L. megaphylla*; Table S2: Primer sequences of candidate reference genes in *L. megaphylla*.
