# Supplementary material for: Selection and Validation of Optimal RT-qPCR Reference Genes for the Normalization of Gene Expression under Different Experimental Conditions in Lindera megaphylla
Source: Plants (Basel). 2023 May 31;12(11):2185. doi: 10.3390/plants12112185 (PMC10255795; doi:10.3390/plants12112185)
Supplement: Supplementary file 1 [file plants-12-02185-s001.zip › plants-2370081-supplementary.pdf]

Supplementary Table S1 The cDNA sequences of the 20 candidate reference genes from *L. megaphylla*

| **Number** | **Gene name** | **Gene sequence** |
|---|---|---|
| 1 | *TCTP* | ATGGGCAAGGTACAAAAATGTTGGATCAGTGGCACCTTCCTTGTAGTAGGCAAACACCACGCTCCCATCATCATGCATG<br>CTCTCACCGACAAAAAATTGAAAATCTTTGAGCTTTGGGAGCAGGAACTTTGTTGCTGCCTCAATGTTCTTCTTGAATT<br>CCTCTTGTTTCTCACCCTCCAACTTAGGTGTCAACAGCTTGATGTACCGTTTCATCCAAGTCACAAACTGCTTCTTGTC<br>AAAAGCGGGTTGTTCCTGGAGGCGAAATGTGTCTACAATATCAACAACTTTTACAGCCTGGTCATCGACTCCCTCGTCT<br>TCATCACCACCTTCCGCAGAAGGGTTTGCCCCTATGTCAACATCAACGGCACCTTGAACAACCCACTTTCCCTCAACTT<br>CCCATAG |
| 2 | *ACT7* | ATGGCTGATGCAGAGGACATTCAACCCCTTGTTTGTGACAATGGAACTGGAATGGTTAAGGCTGGATTTGCTGGAGAT<br>GATGCTCCAAGGGCGGTTTTCCCTAGCATTGTGGGTCGGCCTCGACACACAGGCGTTATGGTTGGGATGGGGCAGAAA<br>GATGCATATGTAGGAGATGAGGCTCAGTCTAAGAGAGGTATTCTTACTTTGAAATATCCCATCGAGCATGGAATTGTGA<br>GCAACTGGGACGACATGGAGAAGATTTGGCATCATACCTTCTACAATGAGCTCCGTGTGGCTCCTGAAGAACATCCAG<br>TTCTCTTGACTGAGGCCCCTCTCAACCCAAAAGCCAACAGGGAGAAGATGACACAGATAATGTTTGAGACTTTCAATG<br>TTCCAGCAATGTATGTAGCCATTCAAGCTGTCCTATCTCTATATGCCAGTGGCCGTACCACAGGTATTGTTCTGGACTCG<br>GGTGATGGTGTTAGCCACACGGTCCCAATCTATGAAGGTTATGCACTGCCGCACGCTATTCTCCGGCTTGACCTTGCTG<br>GACGGGATCTTACAGACTGCCTGATGAAGATCCTTACAGAGAGAGGGTACTCGTTCACTACCACTGCTGAGCGGGAAA<br>TTGTTCGTGACATGAAGGAGAAGCTTGCCTATGTGGCCCTAGACTACGAGCAAGAGCTAGAGACTTCAAAGAGTAGCT<br>CATCAATTGAGAAGAGCTATGAGCTACCTGACGGTCAGGTCATCACCATCGGGGCAGAGCGGTTCCGTTGTCCAGAGG<br>TGCTTTTCCAGCCCTCTCTCGTTGGGATGGAAGCAGCAGGAATCCATGAGACCACATATAACTCGATCATGAAGTGTGA<br>TGTCGATATCAGAAAGGATTTGTATGGCAACATTGTGCTCAGCGGTGGCTCAACAATGTTCCCAGGCATTGCCGATCGA<br>ATGAGCAAGGAGATCACCGCTCTTGCCCCAAGTAGCATGAAGATCAAGGTGGTGGCACCCCCTGAGAGGAAATACAG<br>TGTATGGATAGGGGGCTCTATCTTGGCATCCCTCAGCACCTTTCAGCAGATGTGGATTGCAAAGGCAGAGTACGATGAA<br>TCTGGGCCGTCAATTGTTCACAGGAAGTGCTTTTGA |
| 3 | *GAPDH* | ATGTTTGTTGTGGGTGTCAACGAGAAGGAATACAAGCCTGCCATTGACATTGTATCAAATGCTAGCTGCACGACCAATT<br>GTCTTGCTCCATTAGCCAAGGTGATCAATGATAGGTTCGGCATTGTTGAGGGTTTGATGACCACTGTCCACTCCATTACT<br>GCCACACAGAAAACTGTTGATGGGCCATCCAGCAAGGACTGGAGAGGTGGAAGAGCAGCTGGCTTCAACATCATTCC<br>TAGCAGTACTGGTGCTGCCAAGGCTGTAGGGAAAGTGCTGCCTGCATTAAATGGAAACTTACAGGAATGGCTTTCCG |

| | | |
|---|---|---|
| | | TGTTCCGACGGTGGATGTGTCAGTGGTGGATCTCACTGTGAGACTTGAGAAGGGGGCCACTTATGATGAGATCAAAGC<br>TGCAATCAAGGAGGAGTCTGAGGGCAAGCTGAAAGGAATCTTGGGCTACACGGAGGATGATGTGGTTTCTACTGACTT<br>TGTGGGCGACAGCAGGTCAAGCATATTTGATGCCAAGGCAGGAATTGCACTCAATGACCACTTTGTCAAGCTTGTCGC<br>TTGGTACGACAACGAGTGGGGCTACAGCTCTCGTGTCATTGACCTGATCCGTCACATGGCATCCACTTGTTAG |
| 4 | *UBC36* | ATGGCGAACAGCAACCTTCCTCGCAGGATAATCAAGGAGACGCAGCGGCTCCTCAGCGAACCAGCTCCAGGTATTAGT<br>GCGTCTCCTTCTGAAGACAACATGCGTTATTTCAATGTTATGATTCTTGGTCCAACACAATCTCCTTATGAAGGAGGAGT<br>TTTCAAATTGGAACTATTTTTGCCTGAAGAATATCCAATGGCTGCCCCGAAGGTTCGATTTCTCACAAAAATATACCATC<br>CTAACATAGACAAGCTAGGAAGAATATGCCTTGATATCTTAAAAGATAAATGGAGTCCTGCTCTTCAGATTCGAACAGT<br>ATTACTGAGCATACAGGCACTTCTTAGCGCTCCAAACCCGGATGACCCTCTTTCTGAAAACATTGCGAAGCATTGGAA<br>AACAAATGAAGCCGAAGCTGTGGAAACAGCAAAAGAATGGACCCGTCTATATGCAACTGGGGCATGA |
| 5 | *UBC7* | ATGGCAGCATCGACCCAAGGAAGCCTCCTCCTTCACAAACAGCTCAAAGATCTCATGAAAAACCCGGTCGATGGGTTC<br>TCGGCCGGGCTCGTCGATGATTCGAACATCTTCGAATGGAGCGTAACGATCATCGGGCCGCCTGATACGCTGTATGATG<br>GGGGTTATTTAATGCCATCATGAGCTTCCCAGCAAATTACCCGAACAGTCCTCCATCTGTGAGGTTTATATCGGAAATG<br>TGGCATCCTAATGTTTATCCCGACGGACGTGTTTGCATTTCAATTCTTCATGCCCCTGGGGAAGACCCAAATGGTTATGA<br>ACTAGCAAGTGAGCGTTGGACGCCAGTCCATACGGTTGAAAGCATAGTTTTGAGCATTATTTCTATGCTTTCAAGTCCT<br>AATGATGAATCTCCTGCAAACATTGAAGCTGCAAAAGAATGGAGAGAGCGAAGGGATGAGTTCAAAAAGAAAGTAAG<br>TCGATGTGTCAGACGTTCACAAGAGATGCTTTGA |
| 6 | *EF2* | ATGGTGAAATTTACAGCAGAGGAGCTCCGTAGGATCATGGATTTGAAGCATAATATTCGTAATATGTCCGTTATTGCCCA<br>TGTTGATCATGGCAAGTCCACCTTGACTGATTCATTGGTTGCGGCCGCTGGTATTATTGCACAAGAAGTTGCAGGAGAT<br>GTTCGGATGACCGATACACGCGCGGATGAAGCAGAGCGTGGCATCACAATCAAATCTACTGGCATCTCTCTTTACTATG<br>AGATGGCAGATGAAGAAGTAAAGAATTTCAAGGGTGACAGGATGGGGAATGAGTATCTGATCAACCTCATCGATTCCC<br>CCGGGCATGTTGACTTCTCTTCTGAGGTCACTGCAGCCCTACGTATCACAGACGGTGCACTGGTTGTGGTAGATTGCAT<br>TGAAGGTGTCTGTGTCCAAACTGAAACAGTTCTCCGACAAGCCTTGGGAGAAAGGATCAGGCCAGTCTTGACTGTGA<br>ACAAGATGGATCGTTGCTTCCTTGAGCTTCAGGTTGAAGGAGAGGAAGCCTATCAGACCTTCCAACGGGTCATTGAAA<br>ATGCCAACGTCATCATGGCTACATATGAAGATCCTTTACTTGGTGATGTTCAGGTCTATCCTGAGAAAGGAACTGTTGCT<br>TTCTCTGCCGGTCTACATGGTTGGGCCTTCACTCTCACTAACTTTGCAAAAATGTATGCGTCCAAATTTGGGGTTGATG |

| | | |
|---|---|---|
| | | AAACCAAGATGATGGAGAGGCTTTGGGGTGAAAATTACTTTGATCCAGCTACCAAAAAATGGACGAGCAAGAATACT<br>GGTTCCCCTACATGTAGGCGGGGCTTTGTTCAATTTTGCTATGAGCCCATCAAGCAGATCATCAACATATGCATGACCGA<br>TCAGAAGGACAAACTATGGCCTATGCTACAAAAGCTTGGTGTCACCATGAAGTCCGACGAGAAGGAACTGATGGGGA<br>AGGCTTTGATGAAGCGGGTCATGCAGACTTGGCTGCCAGCTAGCAAGGCTTTGCTAGAAATGATGATCTTCCACCTCC<br>CATCGCCTGCCAAGGCACAGAGGTATCGCGTGGAGAACCTGTATGAGGGACCTCTTGACGACATATATGCAAATGCTAT<br>CAGGAACTGTGACCCTGAAGGGCCTTTGATGCTTTATGTTTCAAAGATGATCCCAGCAGCGGATAAGGGTAGGTTCTTT<br>GCCTTTGGAAGGGTGTTTTCCGGGAAGGTTGCAACTGGTTCAAAGGTGAGAATCATGGGGCCTAACTATGTTCCTGGC<br>CAGAAGAAGGATCTATATGTGAAGAGTGTGCAGAGAACTGTTATTTGGATGGGAAAGAAGCAAGAATCAGTGGAGGA<br>TGTGCCTTGTGGCAACACTGTTGCTTTGGTCGGTCTCGATCAGTACATCACCAAGAATGCAACTCTGACTAATGAGAA<br>GGAAGTCGATGCCCACCCAATCCGTGCAATGAAGTTTTCCGTTTCACCTGTTGTCCGTGTTGCCGTGCAATGTAAGGTT<br>GCTTCTGACCTACCAAAGCTTGTTGAAGGTCTGAAACGATTGGCCAAATCTGACCCTATGGTCGTGTGCACAATTGAG<br>GAGTCGGGTGAGCATATCATTGCAGGTGCTGGAGAACTTCACCTTGAGATCTGCTTGAAAGATTTGCAGGAGGATTTC<br>ATGGGTGGGGCAGAGATTACCGTGTCTCCACCTGTTGTCTCTTTCCGTGAGACGGTGCTTGAGAAATCTTGTCGGACA<br>GTAATGAGCAAGTCTCCTAACAAACACAATCGTTTGTACATGGAAGCTCGCCCCATGGAGGATGGTCTAGCTGAGGCT<br>ATTGATGATGGCCGCATTGGACCAAGAGATGACCCGAAAGTCCGTTCTAAGATCCTATCAGAGGAATTTGGGTGGGAC<br>AAAGATCTTGCCAAGAAGATATGGTGTTTTGGACCAGAAACCACTGGACCCAACATGGTTGTTGATATGTGTAAAGGA<br>GTTCAATATCTTAATGAAATCAAGGATTCTGTTGTGGCTGGATTTCAGTGGGCGTCGAAAGAAGGTGCGCTGGCTGAA<br>GAAAATATGCGGGGGATATGCTTTGAGGTGTGTGATGTGGTGCTTCATGCTGATGCAATCCATAGAGGTGGTGGACAGG<br>TTATTCCAACAGCAAGACGGGCTATCTATGCATCCCAACTGACGGCCAAACCAAGGCTGTTTGAGCCCGTTTATTTAGT<br>GGAGATTCAAGCCCCTGAACAGGCCCTTGGTGGTATCTATGGCGTCCTTAACCAGAAGCGAGGCCATGTATTTGAGGA<br>GATGCAGAGGCCTGGCACTCCACTCTACAACATCAAGGCATACCTGCCAGTTATTGAATCATTTGGGTTCTCGAGTACT<br>CTTAGAGCTGCTACCTCTGGTCAGGCCTTCCCCCAGTGTGTTTTCGATCACTGGGATATGATGTCATCTGATCCAATGGA<br>CCCAAGCACGCAGGCAGGACTGTTGGTTGCTGACATTCGCAAGAGGAAGGGTCTCAAGGAGCAGATGACCCCTCTTT<br>CCGAGTTTGAAGATAAGCTGTAA |
| 7 | *CYP20-2* | ATGGCTACGACTTCTTCTTCCTTCGCGACGACAGTTGCCAACATAAGCTCCACTGCTTCGGATCCCCGAACTCGCTTC<br>CGTCGCCAAGCTCTCAACTCGCCTTCCAACTCACTCAAATTACGAAATTGCCCCTTGCCGACATTGGCGGCTCTGTCA |

| | | |
|---|---|---|
| | | TCGTCGTCGGTGTTGTTCTCCGGCTTCTCAGCATCGCCTCCTCTTCTTCGACGTTCCAAGAGAAGTGGTTTCGTCAGCT<br>CCGTTCGAGCTGCGGCGCAGGAGACAGAATTGCAATCCAAAGTAACACAGAAAGTGTACTTTGACATAAGCATTGG<br>CAACCCTGAGGGACAACTTGTTGGGAGGATTGTGATTGGATTGTTTGGTGATGACGTCCCCCAAACAGCTGAGAACT<br>TCCGTGCTCTATGCACAGGAGAGAAGGGGTTTGGGTACAAGGGATCTACGTTCCATCGTGTTATTAAGGATTTCATG<br>ATCCAAGGTGGAGACTTTGACAAAGGAAATGGAACTGGAGGTAAAAGCATTTATGGTCGTACATTCAAAGATGAAA<br>ATTTCAAGTTGTCTCATGCCGGCCCTGGGGTTCTTAGTATGGCAAATGCAGGACCCAACACCAACGGTAGCCAATTTT<br>TCATTTGCACTGTCAAGACACCTTGGTTGGACCAGAGGCACGTTGTATTTGGGCAGGTTCTGGAGGGCATGGATATT<br>GTCAAGCTGATTGAGTCGCAAGAGACTGACAGAGGAGACCGGCCAAAAAAGAAGGTGGTGATTAGCGACTCTGGAG<br>AGCTCCCTATGGTCTGA |
| 8 | *UBQ* | ATGGTCGCACCCTCGCAGACTACAACATCCAGAAGGAGTCGACCCTCCACCTCGTCCTCCGCCTTCGTGGAGGTATG<br>CAGATCTTCGTTAAGACTCTCACGGGCAAGACCCCGTGGAGGAATGCAAATCTTCGTGAAGACCCTCACGGGCAAGA<br>CAATCACGCTCGAGGTCGAGAGCTCCGACACCATCGACAATGTGAAGGCGAAGATCCAAGACAAGGAGGGGATCCC<br>ACCAGACCAGCAGAGGTTGATCTTCGCTGGTAAACAGCTGGAGGATGGAAGGACCCTCGCCGACTACAATATTCAG<br>AAGGAGTCTACCCTCCATTTGGTGCTGCGTCTCCGCGGTGGGATGCAGATCTTCGTCAAAACCCTGACGGGGAAGAC<br>GATTACTCTGGAGGTGGAGAGCTCTGATACGATTGATAATGTTAAGGCGAAAATTCAGGATAAAGAGGGGATTCCTC<br>CTGACCAGCAGAGGCTGATTTTTGCTGGGAAGCAGTTGGAGGATGGAAGGACTTTGGCGGATTATAACATCCAGAAG<br>GAGTCTACCCTTCACCTTGTCCTCCGTCTCCGTGGTGGTGTCTTCTGA |
| 9 | *TUA* | ATGAGGGAGATCATCAGCATCCACATCGGCCAAGCCGGGATTCAGGTCGGGAATTCGTGTTGGGAGCTGTATTGCCTC<br>GAACATGGCATTCAACCTGATGGAATCATGCCTAGTGATAATACGATAGGTGTTGCACACGATGCTTTCAATACCTTTTT<br>CAGTGAGACCGGGGGAGGGAAGCATGTGCCAAGAGCCATATTTGTTGATCTGGAGCCCACTGTCATTGATGAAGTTAG<br>AACTGGGGCTTATAGGCAGCTTTTTCATCCAGAACAGCTTATTTCTGGGAAGGAAGATGCTGCTAACAATTTTGCCAGA<br>GGACATTATACTGTTGGCAAGGAAATTGTAGATTTATGCCTTGATCGAGTAAGGAAATTGGCAGACAATTGCACTGGTT<br>TGCAAGGGTTTTTGGTCTTCAATGCTGTCGGTGGTGGAACTGGTTCTGGTTTGGGTTCTTTGCTGTTAGAACGTTTGTC<br>TGTTGATTATGGAAAGAAATCAAAGCTTGGGTTCACCATTTATCCTTCTCCACAGGTTTCTACAGCAGTTGTGGAGCCT<br>TACAACAGTGTGCTTTCTACCCATTCCCTTCTTGAGCACACAGATGTTGCGGTTCTTTTGGACAATGAAGCCATTTATGA<br>TATCTGCCGTCGATCTCTAGATATCGAGAGACCAACCTACACCAACTTGAATAGGTTGATATCTCAGATTATCTCCTCGT |

| | | |
|---|---|---|
| | | TGACTACTTCTTTAAGGTTTGATGGAGCAATCAATGTCGATATTACAGAGTTTCAGACCAATCTTGTCCCATATCCACGA<br>ATCCATTTCATGCTTTCCTCATATGCTCCTGTAATCTCTGCTGAGAAAGCATACCACGAACAGATCTCTGTTCCTGAGAT<br>CACGAATGCAGTGTTTGAGCCTTCAAACATGATGGCAAAATGTGATCCTAGGCATGGGAAATATATGGCCTGCTGTTTG<br>ATGTACCGTGGAGATGTTGTGCCAAAGGATGTGAATGCTGCTGTCGCAACAATCAAAACCAAGAGAACTGTTCAGTTT<br>GTTGACTGGTGCCCAACAGGTTTCAAATGTGGAATCAACTACCAGCCCCCAACTGTGGTCCCGGGGGGTGATCTGGCC<br>AAGGTCCAGCGTGCTGTGTGCATGATCAGTAACAACACTGCAGTGGCTGAAGTGTTCTCACGCATCGACCACAAGTTC<br>GATCTCATGTATGCCAAGCGTGCATTTGTTCACTGGTACGTGGGAGAAGGGATGGAAGAGGGAGAGTTCTCAGAAGCC<br>CGTGAGGACCTGGCTGCTCTTGAGAAAGACTATGAGGAGGTTGGGGCTGAGGGTGTTGATGACGTTGATGAAGGCGA<br>AGATTATTGA |
| 10 | *UBC28* | ATGGCATCGAAACGGATTACAAAGGAATTGAAGGACTTGCAGAAGGACCCTCCTGCATCTTGCAGTGCTGGTCCTGTT<br>GCTGATGACATGTTCCACTGGCAAGCAACAATTATGGGACCAGCAGATAGCCCCTATGCAGGTGGTGTATTTCTGGTAA<br>CCATTCATTTCCCCCCAGATTACCCATTCAAGCCACCCAAGGTTGCTTTCCGTACCAGAGTTTTCCATCCAAACATCAAC<br>AGCAATGGTAGTATCTGCCTTGACATTCTGAAGGAGCAGTGGAGCCCTGCTCTAACTATTTCCAAGGTTCTATTGTCAA<br>TCTGCTCCCTGCTGACAGATCCGAACCCTGACGATCCATTGGCACCAGAGATTGCTCATATGTACAAGACTGACCGTGC<br>CAAGTATGAGTCAACTGCACGATCATGGACCCAAAAATATGCAATGGGTTAA |
| 11 | *ICln* | ATGGGTCTAGGTTTACAACTCGTAACGGAACGTATCGGAGACGGCAACGGCCAGCCGTTTCTCGACGCCTCCACCGGC<br>GAGGAACTGATGCTCGTCCAGCCGTCCGTTGCCATCGTGCTCGGAAACCGCCAGCCGGAATCGCCCGGCACCCTCTAC<br>ATCTCCACCAAGAAAGTGGTTTGGTTGAGCGACACCGATAGAGAAAAGGGTTATGCCGTTGATTTCTTATCGGTTTCGC<br>TGCATGCTGTTTCTAGAGATCCTGAGGCTTACGCCTCTCCTTGCATTTATACGCAGATCGATGTTGGTGAAGATGAGGA<br>ATCAGAAGGCTCAGATTCAGAATGTAATGAAAACCTAGAGCTGTCAAAGATAACTGAGATGCGACTAGTGCCATCAGA<br>TCCTAACCAATTGGATACTCTCTTCGAAATCTTCTGTGAATGTGCTGAGTTAAATCCCGAACCTGTTGAAGAGACTGGT<br>GAAGAAAATGAATGGTTTTTTGGTGTTGAACAGATGCAAGACAATACCAATGCAGGGGAAGATTCTGTGTGGCATTTC<br>TCTGAAACTACTGCCAACCCGATCGGTTATGTGAATGGGGATCATGAGCTGGCCCGTACTGGAATTCTTGAGCTTCGAA<br>TTGATGATGAACGTTTCGAGGATGCAGAGGAAAGTGAAGGCGAGATGCACCGTGGCCATTAA |
| 12 | *ubiqunone* | ATGACGGAGGCGATGATAAGGAAGAAGCCGGGGATGGTGAGCGTCAAGGAGATGCCGGTTCTCCAGGACGGCCCTCC<br>GCCCGGCGGCTTCCCTCCGGTCCGCTACGCCCGCCGCATTCCGACCAAAGGCCCCAGCGCCATCGCCATCTTCCTCGC |

| | | |
|---|---|---|
| | | CACCTTCGGCGCCTTCTCATGGGGCATGTACCAGGTCGGCAAGGGCAACAAGATCCGACGGGCGATTAAAGAAGAGA<br>AATATGCTGCTCGCAGAGCAATACTTCCCTTGCTTCAGGCTGAAGAGGATGAAAGATTTGTCCGGGAATGGAAAAAGT<br>ATCTGGAAGAAGAGGCTAGAATAATGAAAGATGTGCCTGGTTGGAAAGTTGGTGAAAACGTGTATCATTCTGGGAAGT<br>GGATGCCACCTGCTAGTGGTGAGCTCCGCCCTGACATCTGGTGA |
| 13 | *PPR* | ATGGAGGCAATCCAATCCCTTTCTCTGCTTTCCTTCTCCTCTAATCTAAGTAAAACAAAGGTATTTGGAACTCCATCCTT<br>CCCCAAGATTAAATTCAATGGAAGAATAGATATAGTTTCTCGTGGTATGTTGACCCCTAGGAAGTTCATGCAGAGGAGA<br>AAGAAAGTGGAAGTGTTTAAGGATGCAGCTGATGAAGCAGACCAGAAGAACTGGAAGAGATTAATGAAGGAAATTG<br>ATGAATCGGGATCGGTAGTTTCGACCCTCAGAGCCCAAAGGACCAAAGGACAGGCCCTTCCTAAAGACATTGTTCTGC<br>GTACTCTTATGAAATTCAAGAAACTAAAGAAATGGAACATTGTCAGTGAGATTCTCGAATGGCTCAGGTGTCAGCATTG<br>GTGGGACTTCAATGAGTTGGACTTTCTACTGCTCATTACAGCTTATGGAAAGCTAGGGGATTTCATTAGGGCGGAAAGA<br>GTTCTAAAGTATATGAACAAACATGGTTATCCGCCAAGTGTGATCTCCTACACAGCCCTTATGGAAGCATATGGACAG<br>GAGGCCAGTATAATCAGGCAGAATCAATATTCCGAAGGATGCAATCTTCTGGTCCTGAACCCTCACCCTTGACATATCA<br>CATAATGCTCAAGATTTGTGTGAAGGGAAACAGATATAAAGAAGCTGAGGAAATATTTGAGACTTTATTGAATGATGAG<br>ATGTCATGCTTTAAGCCAGACCAGCAAATGTTCAATTTGATGATATACATGTATGGGAAAGCAGGAAGCTATGATCAAG<br>CCCGTAAAATATTTGGAAAGATGGCTGAAAGAGGAATACCCAGATCTGCACGCACTTTTAATAGCCTAATGTCATTTGA<br>AACAAATTACAAGGAGGTTTCGAGTATCTATGACCAGATGCAAAGAGCTGATATTCAACCTGATGTTGTGAGCTATACC<br>TTTCTCATAAGAGCATATGCTAAAGCTAGAAGGGAAGAAGAAGCACTGGCAGTTTTTGAGGAGATGCTGGATGCCAGT<br>GTCAGGCCAACTCTTGATGCTTATAACACTTTGCTTGATGCATTTGCGATATCTGGAATGGTGGAGCAAGCCCGGACCG<br>TGTTAAAGAGCATGAGAAGAGACAGGTGCACTCCTAACCTTCACTCTTATACCATGATGTTATCCGCTTTTGTCAATGC<br>ATCTGACATGAATGGGGCTGAAAAGTTCTTTCGACAAATAAAGCAAGATGGCTTCAAACCAAATGTTGTGACGTATGG<br>AACACTGATGAAAGGGTATGGTAAGGTTAATAATCTCGAGAAGATGATGAGTGTGTATGAAGAGATGCGGATGCAAGG<br>AATCAAAGCCAACGAAATCATATTCACGACTATCATGGATGCCCATGGAAAGAATGAGGATTTTGGCAGTGCTGTTGTA<br>TGGTTCAAAGAAATGAAAACATCTGAATTTCCACCTGACCAGAAGGCGAAGAACGTACTTCTGTCCTTGGCAAAAAG<br>TGCTGAGGAAAAGAAGGAAGCCGATGAGCTTGTGGCTAATGCTATTCAGGATCTTAGAGAAGAAGAGGTCTGTGATTT<br>TTCTGAATTTAATGATGCTGACAGAGATGATGCAGTGCAGAATTTTGGTCCTTTCGAGGAACAACAAGATGGGTTAGTG<br>TTAACAAACAATGATCACAGCTCAACTGCAGAGATTACAGAAAGTACAGAATTCGACGATGATGATGAAGATGATGAC |

| | | |
|---|---|---|
| | | TACAACCTGTTAGATTTTGTTTCTTTCAAAGATTCTGTACTTGGTTCTTGA |
| 14 | *SDE2* | ATGACCATCTATCAGATCTTCATCAAGCACCTAGACGGGCGGACCAGATGCCTCCACCTCCCATCTCCGACCATCACTG<br>GCTCTTCCCTCAAACAAACCCTCCACTCCTCCACCAAAATCCCCCTCCACTCCCTCCGCCTGATCTCCGGCAACCGCG<br>ACATCTCCGACGACACCCTAATCTCCGCCTCCGGGGACGATGGCCTTTTCCCCTCCTGCACCGTCCTCCTCCGTCTCCG<br>CGGCGGGAAGGGTGGCTTCGGATCTCTCCTCCGTGGCGCCGCCACCAAGGCCGGCCAGAAGAAGACCAACAATTTCG<br>ACGCCTGCGGCGACATGAGCGGCCGTCGGCTCCGGCACGTGAACGCTGAGAGGAAGCTGGAGGAGTGGAAGGCGGA<br>GGCGGAGGACCGGAGGCTGGAGAAGGTCGCCGAGGATTTCTTGAAGAAGAAGGCGAAGTCCGCGAAGAAAAACAC<br>CTCCGGCGGCGATGCGGAGAAGTATGTGGCGAAGTATAGGGAGGATTCGGCCAAGTGCATGGAGGAGGTGGAGGCGT<br>CAGTGCGAGAGTCGTTTGAGTTGTACGAGGCGTCGAAGCGCAAGGTCTTGCCCGCTTCAGGGCCGGGGGCGTCGAAG<br>CGCCTGAAGATTTGGCTGGGAAAAGGAAAGGCAGTGGAGAGTGATAGTGATGAGGACGAAGATGACGAGAATGAGA<br>AATCAGTGGTTCTTGATGATGGGGGTTGTTTGGTTAGGAGTCAGGTCGAAGAAGGGAGTTCAGGTTCAGTTTCAGTTT<br>CTGCTGGTCAATCAGATGGAGAGTCTTCTGGTGGAGGTTCATCAGAGAGCAATTTGGAAGAAGAAAACTGTAATTCCT<br>GCAAGGGAAATTTGGAACTGGGTGAGGTTCTGGGCAGTGCTTGTTGTACTGCTGATGTAGTCTGTCCTGTTGAACTTC<br>AACCTGCATCCGGGACTTGTGATCAGGGATGCTTAGAATTGGGAAAAGGTTCTGGAAGTGAAGATATTAATGTAGGGA<br>TGGAAGGTTCTGGAATTGTAGCAGTTGATGCTGATGTGGAAGGCTCAGGAAGTGAAGCCATTAATGCGGAAAAAAGA<br>GCTGTTGACACAGAGACAGAATCTGCCACTGAACCTTCTCTGGGAATTTGTGAGGGAACAGTGATTCAGAATGCAAG<br>CATTTTCAGTTTGGGGGGACCGTTAAATTTCACCGACTTCAGCTCAGCAGCTGAGATGGAGGTTTTAGGTATGGATAGG<br>CTAAAGGCTGAGTTGCAGGCACATGGATTGAAGTGTGGGGGTACCTTGCAAGAGCGTGCTGCGAGGCTTTTTCTGCTG<br>AAGACAACACCTATTGAAAAGCTGCCTAAGAAGCTGCTTGCCAAACCTACTACCGGAGGTAAAGGGAAATGA |
| 15 | *EIF4A-3* | ATGGCTGCGGCTGCAACTGCAAATGCAAGTGGTTCGGTGGTCCCTCGAAACCGAAACGGTCCGGGGGGGAGGAATGG<br>TCCTGTGGACGACGACAGCTTGGTGTTCGAGACAAGCCCCGGCGTCGAAGCCGTAACGAGCTTCGACCAGATGGGGA<br>TCCGAGACGACCTCCTCCGCGGCATCTACGCCTACGGATTCGAGAAGCCCTCCGCCATCCAGCAGAGGGCCGTCATGC<br>CCATCATCAACGGTCGAGATGTCATCGCCCAGGCACAGTCCGGCACCGGTAAAACCTCCATGATTGCTCTCACCGTCT<br>GCCAGATGATCGACACCTCTACCCGAGAGGTGCAGGCGCTAATACTGTCACCTACGAGAGAACTTGCTTCACAAACTG<br>AGAAAGTAATCCTAGCTATTGGTGATTTCATAAATGTGCAAGCGCATGCTTGCGTTGGTGGAAAGAGTATCGGAGAGG<br>ATATAAGGAAACTGGAGTATGGAGTTCATGTTGTGTCTGGAACTCCTGGTAGAGTTTGTGATATGATCAAGAGGAGAAC |

| | | |
|---|---|---|
| 16 | *helicase-15* | ATTGCGTACCAGAGCTATCAAATTACTTGTTCTTGATGAATCTGATGAGATGCTGAGCCGAGGATTCAAGGATCAGATAT<br>ATGATGTTTACAGATATCTGCCGCCCGAGCTTCAAGTTGCGTTGATATCTGCCACCCTGCCTCATGAAATTTTGGAGATT<br>ACAAACAAGTTCATGACTGATCCCGTTAGGATACTTGTGAAGCGTGATGAATTGACGTTGGAGGGCATCAAACAATTC<br>TTTGTTGCGGTTGAGCGAGAAGAATGGAAATTCGATACTCTGTGTGATCTTTATGATACGCTTACGATTACCCAAGCTGT<br>TATTTTCTGCAATACGAAAAGAAAGGTGGATTGGTTGACAGAAAAGATGCGTAGCAATAATTTTACGGTCTCTTCCATG<br>CATGGTGACATGCCTCAGAAAGAACGAGATGCAATAATGGCAGAGTTCCGATCGGGTACCACTCGTGTTCTAATCACA<br>ACAGATGTGTGGGCAAGAGGTCTTGATGTTCAGCAGGTGTCTCTGGTGATCAACTATGATCTTCCTAACAATCGAGAGC<br>TTTACATTCATCGGATAGGTCGCTCTGGCCGTTTTGGGCGTAAGGGTGTGGCAATCAACTTCGTGCGCAGTGACGATAT<br>CAGGATTCTAAGAGATATAGAACAGTATTACAGTACACAAATTGATGAAATGCCGATGAATGTAGCTGATCTTATATGA<br>ATGGTGGAAGCGAGGGACAACAACGAAGCTTACGAGGAGGAGCTTCTCGACTACGAAGAGGAGGAAGAAAAGGCCC<br>CCGATTCCGTTGCAGCGAAGGCCGCGGGAGAACCGGTCAAGAAGGGCTATGTTGGAATTCACAGTTCAGGATTCAGA<br>GACTTCCTTTTGAAACCAGAGCTACTTCGTGCTATTGTAGATTCAGGATTTGAGCATCCTTCAGAAGTGCAACATGAAT<br>GCATCCCTCAAGCCATCTTGGGAATGGATGTCATCTGTCAAGCAAAGTCCGGGATGGGTAAAACTGCTGTTTTTGTTCT<br>CTCAACCCTGCAACAGATTGAACCTGTTGCAGGTCAAGTTGCTGCACTTGTTTTATGCCATACAAGAGAGCTAGCGTAC<br>CAGATCTGTCATGAATTTGAGAGGTTTAGCACCTACTTACCTGATATTAAGGTTGCTGTATTCTATGGTGGCGTCAACAT<br>TAAAATTCACAAGGACTTACTTAAGAATGAGTGCCCGCATATCGTTGTTGGGACACCTGGGAGAATACTGGCACTGGC<br>AAGAGACAAGGACTTAGCCTTGAAGAATGTGAGGCATTTTATTCTTGATGAATGTGACAAGATGCTTGAGTCACTTGA<br>CATGCGGAGAGATGTGCAGGAGATTTTTAAAATGACCCCTCATGATAAGCAAGTTATGATGTTCTCTGCAACACTGAGC<br>AAGGAGATCCGACCTGTTTGCAAGAGATTTATGCAAGATCCAATGGAAATTTATGTGGACGACGAGGCCAAATTGACC<br>CTGCATGGGCTCGTACAGCACTACATCAAATTGAGCGAATTGGAAAAAAACCGCAAGTTAAATGACCTGCTGGATGCA<br>TTGGACTTCAATCAAGTTGTCATCTTTGTCAAAAGTGTGAGCAGAGCAGCTGAGTTGAACAAGTTACTTGTGGAATGT<br>AATTTCCCATCTATCTGCATCCACTCTGGAATGTCACAAGAGGAGAGATTGACACGCTACAAGGGCTTTAAGGAAGGT<br>CACAAAAGGATTCTTGTAGCGACAGATTTGGTTGGCAGGGGAATAGACATCGAGCGTGTCAACATTGTCATCAACTAT<br>GATATGCCAGATTCTGCCGACACTTACTTGCACAGGGTTGGCAGGGCTGGCCGATTTGGCACTAAAGGACTTGCAATTA<br>CATTTGTGTCATCTGCTTCCGACTCTGACGTTCTTAATCAGGTTCAAGAAAGGTTTGAGGTGGATATAAAGGAGCTTCC<br>AGAGCAAATTGATACCTCTACCTACATGCCTTCTTGA |

| | | |
|---|---|---|
| 17 | *PAB2* | ATGGCGCAGGTCCAGGTCCAGCCGCAGCCCCCGCAGTCTGGTGCGAATGGTGGGGCGAACCAGTTTGTGTCGACGTC<br>GCTGTATGTTGGAGATCTTGAGCCGAACGTCACGGACTCGCAGCTTTATGATCTGTTCAGCCAGCTTGGCCAGGTCGTG<br>TCCGTGCGCGTTTGCAGGGATGTCAACACCCGCCGCTCCCTTGGATATGCCTATGTCAATTACAACAACACGCAGGATG<br>CTGCGAGGGCAATGGATATGCTGAACTTCACGCCTCTCAATAACAAGCCCATCCGTATAATGTATTCTCACCGTGATCCT<br>AGTATTAGAAAAAGTGGGGCTGCTAATATATTTATCAAGAATCTGGACAAGTCACTAGACAACAAAGCACTACATGAGA<br>CATTTTCTATATTCGGTACCATTTTATCTTGCAAGATTGCTACTGATGGATCCGGCCAGTCAAAAGGCTATGGGTTTGTCC<br>AGTTCGATCAAGAGGAGGCTGCCCAAAATGCTATAGAAAAGCTGAATGGAATGTTGATAAATGACAAACAAGTATATG<br>TTGGACCTTTCCTTCGCAAGCAGGAAAGAGACATGTCTACGAACAAGACAAAATTTAATAATGTCTATGTCAAAAATTT<br>CTCAGAGTCGACAACTGAGGAGGATCTGAAGATGGTTTTTGGCGAGTATGGAACAATTACTAGTGTTGTAGTGATGAG<br>GGATGGTGATGGGAAATCAAAGTGTTTTGGATTTGTTAACTTTGAGGATGCTGATGATGCTGCCCAAGCTGTTGAGGAT<br>CTTAATGGGAAGAAATTTGATGATAAAGAGTGGTATGTTGGTAAAGCCCAAAAAAAATCTGAAAGAGAGATGGAGCTG<br>AAAGGGCGGTTTGATCAAAGTATGAAGGAGGCAGTTGACAAATTTCAGGGCGTAAATTTGTATTTGAAGAATCTGGAT<br>GACAGCATTGGTGATGATAAATTACGAGAATTGTTTTCTGAGTTTGGTACAATTACTTCATGCAAGGTTATGCGAGATCC<br>TGGTGGCATAAGCAGGGGATCAGGATTTGTTGCTTTCTCGACTCCTGAAGAAGCATCTCGAGCTCTCTCAGAAATGAA<br>TGGTAAAATTGTTGTTAACAAGCCGCTATATGTTGCGCTTGCACAGCGTAAAGAAGACAGAAAAGCAAGGTTACAGGC<br>ACAATTTTCACAAATGCGGCCAGTTGCAATGCCACCTTCTGTAGCTCCTCGTGTGCCAATGTATCCCCCTGGTGCTCCA<br>CCAATGGGGCAAGTATTTTATGGCCAAGGCCCTCCTGCACTTATTCCTCCACAGCCTGGTTTTGGGTACCAGCAACATC<br>TTGTCCCTGGGATGAGGCCTGGAGGGGCTCCTATGCCAAATTTCTTCGTGCCAATTGTCCCACAAGGTCAGCAGGCTC<br>AGCGCCCTGGTGGGAGACGTGCAGGAGCAGGACCGGTGCAACAAAACCAGCAACCTGTGCCGATGCTTCAGCAGCA<br>GATGCTTCCAAGGGGGGGACGTGTCTACCGGTACCCACCTGGGCGTAACGTGCCAGATGTATCAATGCCTGGTGTAGC<br>TGGAGGCATGCTTTCTGTTCCATATGATATGGGGGCCATGCCATTAAGAGAATCACAGCCTATTCCGATTGGGGCACTGG<br>CTTCAGCTCTTGCAAATGCTACTCCTGATCAGCAGAGGATGATGCTGGGGGAGAGCTTGTACCCACTTGTGGATCAGCT<br>GGAGCATGATATGGCTGCCAAAGTGACAGGAATGCTTTTGGAGATGGACCAGACTGAGGTTCTGCACCTGTTGGAATC<br>CCCGGAGGCTCTTAAAGCTAAAGTTGCTGAAGCCATGGATGTTCTGAGGAATGTTGCTCAGCAGCAGCAGTCTGGTTC<br>CCCGACTGATCAGCTGGTGGCACTTTCTCTGAGTGACACACTTGTTTCCTGA |
| 18 | *CYP95* | ATGAAAGCTGTCAAGGTTGTCTCTTCTGATGCTGATGGCCGTGAAATGGGACGAAGAGGAAAGCACAAGAAACATTC |

| | | |
|---|---|---|
| | | AAAGGAGCAAAGAAGGAAAAGGAGAAGAAAATACTACTCATCTGAATCAGATAGTTCATCAGATACTGAAACAGAAT<br>CATCAGATTATGGCAGTGATTCTGATTCATATTCATCTTCATCATCTTATGTTACTTCTTCAAGTGATGACAGGCATAGAA<br>GGAGAAAGAAAGCATCCAAGAGGGACAAGTATAGGCGTGGCAAAAGTAAAAGGGATAGACGACGTGATAAGAGACG<br>TCGGCGGCATTATAGGAGATCAAAACGCAAGTCAAAACGGATCTTGGAGAGTTCTACTGATACTGAAACCGAGACTAC<br>AAGTGAAAGTAGCTCTGAGGAGAATGGAACTAATGATCGTCAACGAGCTCGGAAGTCTAAACATTCTAAGATGTCAGT<br>TGGGAATCAGTCCGTCTTGGTGGAACAAGAAGCTACAGTTTCTAAGAGAATGAATGGGGATACAATTGATGAGCTTGA<br>ACCAGTGGAGCAAGCATTACCAAGAGAAAATGGAGAGCTTCAAAGCAATGGGATAGAAATGGAAGCTAAGTCTGATA<br>AAAGTCAAGATAGGCGACCTAATTTGGATGATCCTCCAAGCAAATCTAGGAGTCGAAGCCCGAGTCCTAGGGGGAGCA<br>TGAGCAAGAGCATGAGCGTAAGTCCAAGGAGGAATTTGAGCAAGAGCCCAAGTACGAGCCCTCGGGGGAGTGTGGG<br>CAGGAGCCCCACTCCAGGTCACAGCGGAAGTCCTGTGAAAGCACCAAAAGGGGGCAGCGTTAGCAGAAGGCCTTCT<br>AGAAGAAGCACTAGCGCTGGAAGCCCAGCTAGAAGCATTAGTAGAAGCCCAGTTATGTCCCGGCCTATAAGAAGCATT<br>AGCAAAAGCCCTTCAAGAAGCATTAGCAGTGGAAGCCCAGCTAGAAGAAGCATCAGCAGAAGCTCGGTGAGGCCCA<br>AATCTGGAAGAAGCATTAGCAGAAGCCCAGTTAGAGCACGGCCTCAAAGAAGCATTAGAAGGGGTTCAGTGAGGTTG<br>TTTTCACGAAGAAGCGCAAGCCGTAGCCCAGTTAGGGCCCGTTCACGAAGAACACGCAGCAGAAGCCCTGTAAGGGC<br>CTCTCGAATAAGCATTAGTGGGAGCTCAGTTAGGGACAGGAGCATTAGCCGGAGTCCTGTGAAAGCACCTAGCAGAA<br>GTAATCGCCGCAGCTATTCGAGGAGTCCTGTGAGCCATGGGCGCAAGGCACCAACTCCCCGTGATAGGAGCTTGTCAA<br>GAAGTGTTTCTCCAGATGGATCACCCAAGCGCATTCGAAGGGGGAGGGGGTTCAGTCATCGTTACTCTTATGCTCGAC<br>GTTATCGAACCCCATCTCCTGATCATTCTCCTGTAAGGTCTCATCGTTATGGAGGAAGAAGTGAACGCGACAGATATTC<br>AAGTTACAGGAGCTACTATGATCGCTCTCCACCAAGGCGCTATAGGAGCCCTCCAAGGGGAAGAACTCCTCTAAGATA<br>CAGAAGCAGGAGGAGCCGGACTCGAAGCAGAAGCATATCCTGCAGCCCAGTAGGATACCGAGGGCGAGCAAGAGGC<br>TACAGCCAGAGCCCAGTACGCAGCCGTTCCCCATTTGAGAGACGGAGGATCACAGGAGACATATCCCGCAGAGAGAA<br>GAGGTCACCTTCTAGGAGTAGCAGCTCATCACGGTCAAGGTCATCACCACCTGACACCCTGAAGCGGATCAGCAGGG<br>AGAAGTCACGGTCACCATCGAGCAGCCCCTCAGGAAGGAATGGCTTGGTTTCATATGGAGATGGTTCTCCTGATTCTG<br>GGCAAAGGTCGGATTGA |
| 19 | *RHA2A* | ATGGGTCTTTGCAGCGATCTGAACGATGTATCTTCAGATTCAATCCCATTATTCCTTGTAGCTCGATTTGCCAACTGC<br>ATATGTTATCTCAAATCCCTGCTTTTCTGTCTTCCCCAATCCTTGGGGCTCTCTAGATTTGGAGGAGCAGCTGGGGTTG |

| | | |
|---|---|---|
| | | TTTCAGGGTTCGGTCCTTTTGGGTTGGTGGGTTCGGGTCTGGCGGGTCTGGTTCTGCTCGCGGAGCAGCTTAACGTGC<br>ACCGGGTCTTCACCTTTAGCGGGAGCGATGTCGGGCCGGATTGCGTGGTCTGCTTATCGGGGCTGAGAGAAGGGGAG<br>CAAGTGCGCATGTTAGCATGCCGCCATGTCTTCCACAGAGAGTGCTTGGATGGGTGGTTTGACCACCAGAACCTGAA<br>CTGCCCGCTCTGCCGCGCGCCGCTCGTGGCGGAGGAGCGAGTGTCGGAGATGGGCCGGCGCGTGGGTGGCGACCTA<br>GTCACGTGGTTCTCACCGACCCGATGA |
| 20 | *EF1 α* | ATGGAGGACTTGCGACCCGACAACAACGATCTTCATCCTCCTGCCCACGTGGACGGTGGAGAGCCGGACGATTGGAG<br>CAAGGAAGACGATCCAATGGTTGAAGATCGCCCTCCGTCTGCAACAGAAACCCCTGCTCCTTCCTTCCAAGATATGAA<br>GGATGAGGCTCAATCTAGCATTCAATCATTGCAGCTGGAGCCAAAAGGGAAAGATAAGGAAATGAGTACACATGAAG<br>AAGATGAAGAATTGGATGAAAAGGAAGAAAATAATAAGCGTCACCTAAATGTTGTCTTTATAGGTCATGTTGATGCTGG<br>AAAGTCGACAACTGGGGGGCAGATACTCTTCCTCAGCGGTCAGGTTGATGATAGAACAATTCAAAAATATGAGAAAGA<br>AGCCAAGGACAAAAGTAGAGAGAGTTGGTATATGGCATATATCATGGACACAAATGAGGAGGAGCGTGTAAAGGGGA<br>AGACTGTAGAGGTTGGCAGAGCTCATTTTGAGACAGATACTACAAGGTTCACAATTTTGGATGCTCCGGGTCACAAAA<br>GCTATGTCCCTAACATGATCAGCGGTGCATCTCAAGCTGACATTGGAGTTCTGGTTATTTCAGCCCGGAAGGGTGAGTT<br>TGAGACTGGGTATGAAAGGGGTGGACAGACCCGTGAACATGTGCAACTTGCAAAAACTTTAGGTGTTTCTAAGTTGCT<br>TGTTGTTGTCAACAAGATGGATGATCCTACTGTGAAATGGTCTAAAGAAAGGTTTGATGAAATTGAATTGAAGATGACA<br>CCATTCTTAAAATCATCTGGTTACAATGTAAAAAAAGATGTTCAATTTCTTCCAATATCTGGTCTAGTTGGTACTAACATG<br>AAAACTAGAGTGGACAAGAGTTTGTGTGGATGGTGGAATGGTCCGTGTCTTTTTGAAGCACTTGATGCATTGGAAATT<br>CCTCTACGTGATCCAAAAGGCCCATTTAGGATGCCAATAATTGATAAATTCAAGGACATGGGAACAGTTGTTATGGGCA<br>AAATTGAGTCTGGTAGTGTCCGTGAAGGCGACAGTTTGTTGATCATGCCAAATAAGGCTCATGTGAAAGTGATATCCAT<br>ATATTGTGATGAGAGCAAAGTTAGATGTGCTGGCCCTGGTGAAAATGTTCGAGTTAAATTGTCAGGGATTGAAGATGA<br>GGATATTTTGGCAGGTTTTGTCCTTTCAAGTGTTGCCAAGCCGATTGTTGCAGTTACTGAATTTACTGCTCAATTGCAGA<br>TCCTGGAGTTGCTGGATAACGCAATTTTTACTGCTGGTTACAAGGCAGTTTTGCACATTCATGCGATTGTTGAAGATTCT<br>GAGATTGTTGAGCTGCTATCACAGATTGATCCAAAGACGAAGAAACCCATGAAGAAAAAAATTCTTTTTGTGAAGAAT<br>GGCGCTATTGTCGTATGCCGCATTCAGGTGAATAACTTGATATCAATCGAGAACTTTTCCGATTTTCCACAGCTAGGAAG<br>GTTTACCCTTCGTTCTGAAGGAAGAACGATTGCTGTTGGGAAAGTTATATCTCTTCTTCCATGA |

Supplementary Table S2 Primer sequences of candidate reference genes in *L. megaphylla*

| Gene | Forward primer (5'-3') | Reverse primer (5'-3') | Product length / bp | Sequence |
|---|---|---|---|---|
| *LmNAC83* | GAGCAACTGGGTCAGGTTATT | CAGCACCTTCTTCATCCCTAC | 95 | ATGGAGAAAATGAACTTTGTGAGAGATGGAGTTGTGAAATTGCCTCCTGGGTTCCGGTTCCATCCCACTGATGAAGAGCTTGTTGTTCAGTATCTTAAGAGGAAGGTCTTCTCTTGCCCTTTGCCTGCCTCGATTATCCCCGAGATTGACGTTTGCAAGTTCGATCCCTGGGATCTTCCAGGCGATTCGAAGCAAGAGAGGTATTTCTTTAGCATCAGGGAAGCAAAATACCCAAATGGGAATCGACCCAACAGAGCAACTGGGTCAGGTTATTGGAAGGCCACTGGTTTGGATAAGCAGATTGTGGCTTCGAGGAGCGACCAAGTGGTAGGGATGAAGAAGGTGCTGGTTTTCTACAGAGGAAAGCCCCCAAAAGGGTCTAGAACTGATTGGATCATGCATGAATACCGCCTTTCGGACGCCACTGGCAGCAGAGGTTGCTTTGTCCCTCAGAGGAAAAACTCCACCCAAAATGCCATGATGCAAATGGAAGATTGGGTTCTCTGTCGCATCTTTTTGAAGAAAAGAAGTGCTAAAAATGATGGAGAAACCACCCAAGCGTGCACCAATTACAGAGCCAGGAATCTTGGGGTACCTAGAAACGGTTTCATCGATTTCTTGGCGCGCGAGACTTCTTCTTCATCTGATTCAAGCAGTATCACGGAAATCTCTTCAGCTGGATCGCCCAATGAAGAAAACTGCAGCTCCAATAGCTTCTCTTCATCTCCTTGTGAAAGAAAGCCATAA |
| *LmERF60* | GGTTGTTGGAGGAGGTGAAG | CGGCTCATTGAAATCAGGAAAC | 122 | ATGGCACTTCAGAACCTGAGGCAGTGGCAGTTTTTGGGACCCAGGGTCGCACCCATGAAGCAAGTGGGCTCGCCACCCAAACCCACCAAGCTCTACCGTGGTGTACGGCAGCGCCACTGGGGGAAGTGGGTGGCGGAGATCCGCCTCCCCAAGAACCGGACCCGCCTCTGGCTCGGCACCTTCGACACCGCTGAGGAGGCCGCGTTGGCTTACGACAAGGCCGCCTACAAGCTCCGTGGGGAGTTCGCCCGCCTCAACTTCCCCCACCTCCGCTACCAGGAAGCCCCTCTCGGCGGCGAATTCGGCGCCTACCGGCCGCTCCAGTCCTCTGTTGACGCGAAGCTACAAGCTATCTGTCAAAGCTTGGGTAACAACTCGCAGAAACAGGGGAAATCAGAGAGCCCGGTGGCCGCCAAGCAGCCGCAGCCATCTGATGCGTCCAAGCCGGAGATGGGGTTGTTGGAGGAGGTGAAGGTGGAGACCTCAAGCCCGCCGTCGTCCGTGACGTCGGAAAGCGACGAGTCTGGCGGGTATTCGCCGGATTCTGATATCAAGTTTCCTGATTTCAATGAGCCGCCATGGGATGAGTCTGAGAGCTTTCTGTTGCAGAAGTTTCCATCTTTGGAGATTGATTGGGAGGCTATTCTATCCTAA |

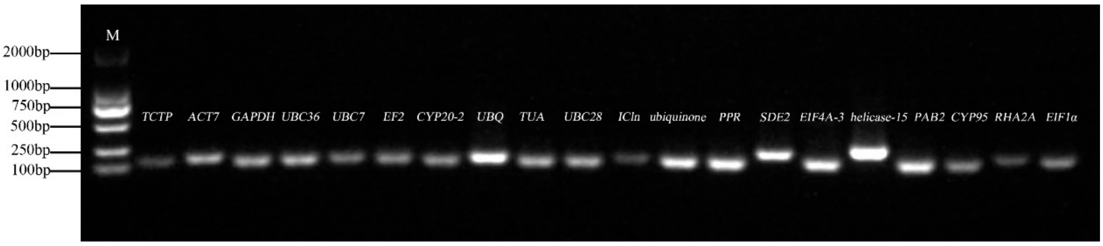


**Supplementary Figure S1.** The PCR products electrophoresis result of the 20 candidate reference genes

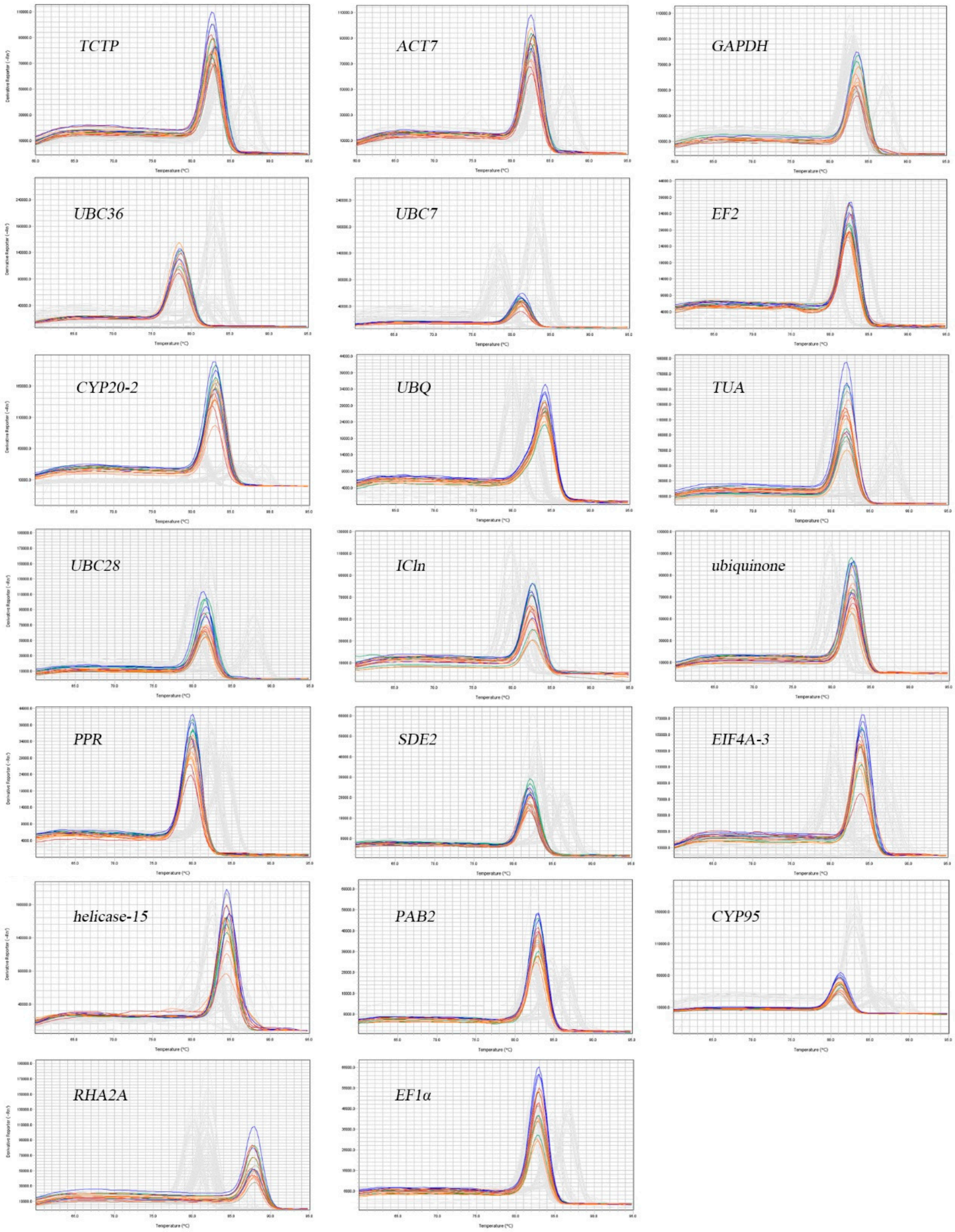

**Supplementary Figure S2.** Melting curves of the 20 candidate reference genes in L. *megaphylla*. The RT-qPCR reaction was performed for 40 cycles, and multiple repeating peaks were generated at the same reaction temperature. The single peak of the melting curves of each candidate reference gene indicated the specificity of the primer.
